# Cyclohexane Oxidative Dehydrogenation on Graphene-Oxide-Supported Cobalt Ferrite Nanohybrids: Effect of Dynamic Nature of Active Sites on Reaction Selectivity

**DOI:** 10.1021/acscatal.3c02592

**Published:** 2023-10-05

**Authors:** Shashikant A. Kadam, Stefania Sandoval, Zdeněk Bastl, Karolína Simkovičová, Libor Kvítek, Juraj Jašík, Joanna Elżbieta Olszówka, Stanislav Valtera, Mykhailo Vaidulych, Jaroslava Morávková, Petr Sazama, David Kubička, Arnaud Travert, Jeroen A. van Bokhoven, Alessandro Fortunelli, Armin Kleibert, Martin Kalbáč, Štefan Vajda

**Affiliations:** † Department of Nanocatalysis, J. Heyrovsky Institute of Physical Chemistry of the Czech Academy of Sciences v.v.i, Dolejškova 3, 18223 Prague, Czech Republic; ‡ Department of Low Dimensional Systems, J. Heyrovsky Institute of Physical Chemistry of the Czech Academy of Sciences v.v.i, Dolejškova 3, 18223 Prague, Czech Republic; § Department of Physical Chemistry, Faculty of Science, Palacký University Olomouc, 17. Listopadu 12, 77900 Olomouc, Czech Republic; ∥ Department of Structure and Dynamics in Catalysis, J. Heyrovsky Institute of Physical Chemistry of the Czech Academy of Sciences v.v.i, Dolejškova 3, 18223 Prague, Czech Republic; ⊥ Department of Petroleum Technology and Alternative Fuels, University of Chemistry and Technology Prague, Technická 5, 166 28 Prague, Czech Republic; # Normandie Univ., ENSICAEN, UNICAEN, CNRS, Laboratoire Catalyse et Spectrochimie, 14000 Caen, France; ∇ ETH Zürich, Vladimir-Prelog Weg 1, Zürich 8093, Switzerland; ○ CNR-ICCOM, Consiglio Nazionale delle Ricerche, via G. Moruzzi 1, Pisa 56124, Italy; ◆ Swiss Light Source, Paul Scherrer Institut, 5232 Villigen PSI, Switzerland; ¶ Department of Mathematics, Informatics and Cybernetics, University of Chemistry and Technology Prague, Technická 5, 166 28 Prague, Czech Republic

**Keywords:** spinel, CoFe_2_O_4_, RGO, cyclohexane, oxidative dehydrogenation, XPEEM, support effects in catalysis, dynamic active sites

## Abstract

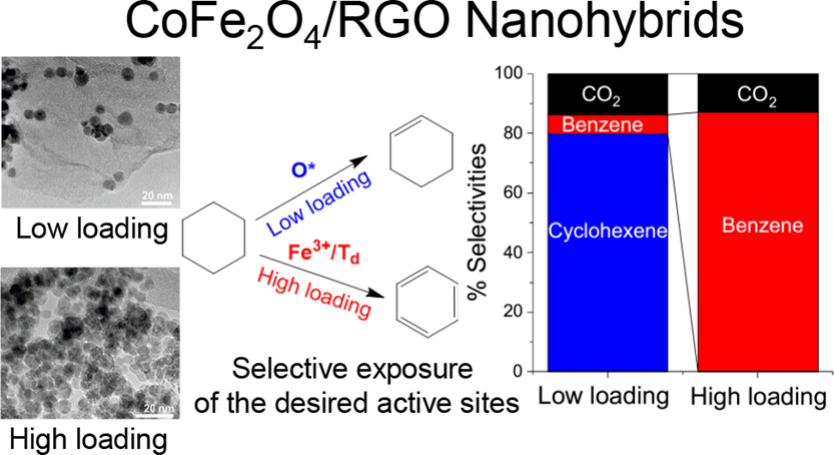

In this work, we investigated cyclohexane oxidative dehydrogenation (ODH) catalyzed by cobalt ferrite nanoparticles supported on reduced graphene oxide (RGO). We aim to identify the active sites that are specifically responsible for full and partial dehydrogenation using advanced spectroscopic techniques such as X-ray photoelectron emission microscopy (XPEEM) and X-ray photoelectron spectroscopy (XPS) along with kinetic analysis. Spectroscopically, we propose that Fe^3+^/T_d_ sites could exclusively produce benzene through full cyclohexane dehydrogenation, while kinetic analysis shows that oxygen-derived species (O*) are responsible for partial dehydrogenation to form cyclohexene in a single catalytic sojourn. We unravel the dynamic cooperativity between octahedral and tetrahedral sites and the unique role of the support in masking undesired active (Fe^3+^/T_d_) sites. This phenomenon was strategically used to control the abundance of these species on the catalyst surface by varying the particle size and the wt % content of the nanoparticles on the RGO support in order to control the reaction selectivity without compromising reaction rates which are otherwise extremely challenging due to the much favorable thermodynamics for complete dehydrogenation and complete combustion under oxidative conditions.

## 1 Introduction

Alkane dehydrogenation reactions have been extensively investigated because of their practical industrial importance.^1,2^ Typically, supported noble metals, metal clusters, and alloys are used as catalysts in alkane dehydrogenation and the major mechanistic underpinnings behind their activity involved metal-support interactions such as the formation of a metal-proton adduct,^3^ charge transfer between the metal atoms and the support,^4^ and polarization of the metal particles by cations of the support present within vicinity.^5^ The oxidative dehydrogenation (ODH) of alkanes on the other hand is attractive because it essentially removes the thermodynamic constraints associated with the nonoxidative dehydrogenation pathway, facilitates C–H bond rupture, and alleviates the catalyst deactivation by coke formation. The ODH reactions, however, undergo thermodynamically more favorable complete dehydrogenation with poor selectivity control to desired intermediates and often lead to total combustion. Therefore, a complete mechanistic understanding and robust catalyst design with precise identification of active sites responsible for total combustion and/or partial/full dehydrogenation along with the ability to limit or fully suppress the combustion channel is receiving considerable attention at present.

Here, we investigate ODH of cyclohexane as a prototypical representative of these fundamental and practical challenges in a strongly structure-sensitive reaction. Besides, the selective production of cyclohexene from partial dehydrogenation of cyclohexane is desirable as it provides an important building block and precursor for the synthesis of many chemicals (e.g., pharmaceutical precursor, adipic acid in the production of nylon, etc.). Usually, cyclohexene production is carried out from partial hydrogenation of benzene using expensive reductants such as H_2_ and noble metal catalysts (e.g., Pt, Pd, Ru, etc.)^6,7^ under energy-expensive conditions^8^ (e.g., high H_2_ pressures, 4–5 MPa). The selectivity toward cyclohexene, however, is limited (only 5–35%), as these conditions often lead to complete hydrogenation to produce cyclohexane. In contrast, the ODH pathway uses inexpensive oxidants such as O_2_ or CO_2_, but partial and selective dehydrogenation is still a challenging task even on the best-performing catalysts such as Au, Pd, V, Co, etc.^9−13^ The resulting loss of primary products not only interferes with rigorous kinetic inquiries necessary for unequivocal mechanistic conclusions but also precludes the efficient use of these catalysts in practice. There is little information on how to selectively desorb the primary dehydrogenated unsaturated product at practical conversions without compromising reaction rates.

The selective desorption of a desired product in a sequential reaction essentially requires decreasing its binding energy to the catalyst surface as previously demonstrated on Co_4_ sub-nanometer clusters deposited on the Al_2_O_3_ support,^14^ transition metal phosphides (TMP)^15−18^ and metal clusters on carbon supports such as graphene and graphene oxide (GO).^19,20^ The GO-derived materials are gaining much attention as they provide an inexpensive alternative for graphene and possess charge transfer ability because of the highly delocalized and solvated electrons. For instance, recent DFT calculations show that the originally localized electrons on PtM (M = Co or Fe) dimers become highly delocalized when these dimers are deposited on nitrogen-doped graphene.^21^ Such delocalization of electrons and redistribution of charge density on the PtM dimer upon introduction of N-doped graphene eventually leads to the loosening of the Pt-H bond, facilitating the desorption of H_2_ in the hydrogen evolution reaction (HER).^21^ Such electronic metal support interaction has been previously reported for several reactions for graphene-derived supports.^22,23^

Starting from these grounds, in this study, we demonstrate a strategy to promote the desorption of the primary dehydrogenation product (cyclohexene) in the cyclohexane ODH reaction and inhibit its concomitant consumption to sequential dehydrogenation and combustion channel. For this purpose, we employ a set of catalysts, prepared by the decoration of GO with spinel oxides (cobalt ferrite, CoFe_2_O_4_) nanoparticles, leading to simultaneous reduction of GO (formation of reduced graphene oxide (RGO)). The novel synthetic approach involves a two-step protocol viz., synthesis of the CoFe_2_O_4_ NPs and their loading onto the GO surface with a highly narrow size distribution. Oxides with spinel structures such as CoFe_2_O_4_ are making a significant impact on research in the field of catalysis because they offer an ability to fine-tune their properties including chemical and thermal stability, desirable band gaps, multiple oxidation states, coordination geometries, etc.^24^ Spinel ferrites with general formula AFe_2_O_4_ have been particularly shown as promising catalyst for oxygen evolution (OER) and oxygen reduction (ORR) reactions,^25,26^ water-gas shift reaction,^27^ complete oxidation of methane,^28^ and CO oxidation.^29^ Cobalt ferrite in its most stable phase has an inverse spinel structure, (Fe^3+^)[Co^2+^Fe^3+^]O_4_, in which tetrahedral sites (T_d_) are occupied by Fe^3+^ and one-half each of the octahedral sites (O_h_) are occupied by Co^2+^ and Fe^3+^, respectively, but an alternative normal spinel structure, (Co^2+^)(Fe^3+^)_2_O_4_, is also relatively stable, in which tetrahedral sites (T_d_) are occupied by Co^2+^ and octahedral sites (O_h_) are occupied by Fe^3+^, respectively. The rich oxidation states and variable coordination geometries make CoFe_2_O_4_ an attractive candidate for O_2_ activation,^30,31^ which is a necessary step for alkane/alkanol oxidation and reduction reactions.^11,32^

Based on the spectroscopic (XPEEM and XPS) and kinetic evidence, we propose that the Fe^3+^ cations at tetrahedral locations (Fe^3+^/T_d_) could be responsible for full cyclohexane dehydrogenation producing benzene while kinetics show that the oxygen-derived species (O*) are responsible for partial dehydrogenation producing cyclohexene. We also reveal that Co and Fe cations can cooperatively migrate and exchange their coordination as a function of reaction conditions and RGO can specifically interact with Fe^3+^/T_d_ species and mask their participation in the reaction. By varying the wt % content and size of CoFe_2_O_4_ nanoparticles on the RGO support, we manage to control the abundance of Fe^3+^/T_d_ and O* species on the nanoparticle surface, thus achieving full control over primary and tertiary dehydrogenation reaction selectivities. To the best of our knowledge, no previous study has shown how to control the selective desorption of a primary product over a thermodynamically favored full dehydrogenated product by manipulating the local environment of the catalyst and the metal-oxide/support interactions, thus tuning the abundance of the respective active sites (Fe^3+^/T_d_ and O* species).

## 2 Experimental Methods

### 2.1 Reagents

Iron(III) nitrate (nonahydrate), cobalt(II) nitrate (hexahydrate), and graphene oxide, were purchased from Sigma-Aldrich. Oleic acid (Sigma Aldrich), ethanol (99.9%, VWR Chemicals), n-hexane (anhydrous, Penta Chemicals Ltd.), acetone (98%, VWR Chemicals), n-pentanol (95–97%, Penta Chemicals Ltd.), and toluene (99.8%, Acros Organic) and were used as received.

### 2.2 Catalysts Synthesis and Characterization

RGO-decorated CoFe_2_O_4_ nanoparticle (NP) samples were prepared by initially synthesizing CoFe_2_O_4_ small and large NPs followed by their attaching onto the GO surface, leading to the reduction of the latter.

#### 2.2.1 Solvothermal Synthesis of CoFe_2_O_4_ Small NPs (S-CoFe)

To synthesize CoFe_2_O_4_ NPs, iron(III) nitrate (16 mmol) and Co(II) nitrate (8 mmol) metal precursors were used. The mixture of these precursors was dissolved in 10 mL of distilled water and added to a sodium oleate solution, previously prepared by dissolving NaOH (2.64 g) in 10 mL of distilled water and subsequently adding 20 mL of EtOH and oleic acid (18.4 g) under magnetic stirring. After mixing both metallic salts and the oleate, a black and viscous solution was obtained. n-Hexane (anhydrous) (20 mL) was added to the above black viscous solution, and the system was refluxed for 1 h and cooled to room temperature. A two-phase (aqueous and organic) system was obtained. The aqueous fraction was discarded, and the organic part was washed by adding 20 mL of water, 5 mL of ethanol, and 5 mL of n-hexane, and the system was boiled for 30 min. The protocol was repeated twice after the removal of the aqueous phase (containing the inorganic residues). Then, 15 mL of 1-pentanol was added to the flask and the n-hexane was removed from the system using a rotary evaporator to obtain the Co–Fe oleate mixture. The Co–Fe oleate mixture (7.89 g) was then mixed with 16.25 mL of 1-pentanol and 10 mL of distilled water and the mixture was transferred into a Teflon liner and stirred for 15 min and then heated in an autoclave under solvothermal conditions during 12 h at 180 °C. After this treatment, the dark fraction was separated from the aqueous phase, and it was subsequently washed by dispersion in n-hexane and EtOH mixtures. The obtained powder was centrifuged, and the supernatant was discarded. The washing protocol was carried out three times to obtain S-CoFe NPs. These S-CoFe NPs were redispersed in 10 mL of n-hexane and stored.

#### 2.2.2 Synthesis of Large Core-Shell CoFe_2_O_4_ NPs (L-CoFe)

In order to obtain NPs with a larger diameter, the smaller CoFe_2_O_4_ NPs obtained in 2.2(a) were used as seeds. Small CoFe_2_O_4_ NPs (75 mg) (after removing n-hexane by evaporation) were then mixed with the Co-Fe oleate mixture (3.28 g). After vigorous mixing with toluene (10 mL), 1-pentanol (8.4 mL), and H_2_O (5 mL), the mixture was transferred into the autoclave, and the system was heated at 220 °C for 12 h. The obtained dispersion was then washed using a similar protocol as described before, and the obtained powder was redispersed in n-hexane and stored.

#### 2.2.3 Decoration of RGO with CoFe_2_O_4_ NPs (RGO-CoFe)

A further solvothermal treatment was employed for obtaining the RGO-CoFe-based catalyst. For this purpose, graphene oxide (GO) was mixed with both small and large CoFe_2_O_4_ NPs, respectively, using pentanol/toluene/H_2_O mixtures to ensure the proper interaction between the precursors. Different CoFe_2_O_4_/GO ratios were tested (Table 1) in order to modify the final NP loading. In the case of small NPs, the reaction was carried out for 12 h at 120 °C, while decoration with large particles was carried out by heating the system at 180 °C.

**Table 1:** Synthesis Conditions Used for the Solvothermal Preparation of Both Unsupported and RGO-Supported CoFe_2_O_4_ NPs

sample	precursor	CoFe/GO ratio	temperature (°C)
S-CoFe	Co-Fe oleate		180
L-CoFe	S-CoFe/Co-Fe oleate		220
RGO-CoFe1	S-CoFe	1.66	120
RGO-CoFe2	S-CoFe	0.41	120
RGO-CoFe3	S-CoFe	0.15	120
RGO-CoFe4	L-CoFe	2.33	180
RGO-CoFe5	L-CoFe	0.41	180

#### 2.2.4 Characterization

High-resolution transmission electron microscopy (HRTEM) was performed using a JEM-2100Plus Electron Microscope (JEOL) for microstructure evaluation. TEM micrographs were acquired using an accelerating voltage of 200 kV. NP size distribution was determined by measuring ca. 200 specimens using the ImageJ processing software. Samples were prepared by dispersing a small amount of powder in n-hexane and placed dropwise onto lacey carbon TEM grids. In the case of self-standing NPs, the grid was covered by an ultrathin carbon layer (Cu, 300 mesh, SPI supplies) (SI, Section S1).

Thermogravimetric analyses (TGA) were performed on a Netzsch instrument, model STA 449 F1 Jupiter, under flowing oxygen at a heating rate of 10 °C min^–1^. The experiments were performed under a flow of pure O_2_ (40 mL/min, Messer) using Ar as a protecting atmosphere (20 mL/min, Messer) (SI, Section S2).

### 2.3 Cyclohexane ODH Reaction Conditions, Product Identification, and Steady-State Catalytic Rate Measurements

A microcapillary reference reactor was used for steady-state catalytic rate measurements. The reactor consists of a quartz capillary tube (∼1 mm OD, thickness ∼ 0.4 mm, and length ∼ 50 mm) and two furnace pieces (30 mm long) holding resistive ceramic heaters placed immediately on each side of the capillary providing ideal plug-flow catalytic reactor characteristics with reactant residence times in the range of 1–30 s. The reaction temperature was controlled by an electronic temperature controller (Eurotherm 2404) and Kepco power supply and measured by using an in-bed thermocouple (type K, Omega) with an accuracy within ±2 °C. Catalyst aggregates (<0.0005 g with bed length ∼1 to 2 mm) were held on a porous quartz wool bed within the capillary reactor. Catalysts were treated in flowing O_2_ (500 cm^3^ g^–1^ s^–1^, 4030 ppm in He, Messer) by heating to 400 °C (with a temperature ramp of 5 °C min^–1^) and holding for 2 h before cooling to reaction temperature. This treatment was monitored by mass spectrometry. Cyclohexane (3914 ppm in He, Linde) and O_2_ were delivered through gas cylinders into the heated transfer lines (70 °C). All the gas flow rates were metered by using mass flow controllers (Brooks Instruments). The partial pressures were adjusted by dilution in He (99.999% Messer). The identity and concentrations of reactants and products in the reactor inlet and effluent streams were determined using gas chromatography (Inficon MicroGC Fusion) equipped with Rt-Molsieve 5A (0.25 mm ID 10 m), Rt-Q Bond (0.25 mm ID, 12 m), and Rxi-1 ms (0.15 mm ID, 20 m) columns and Thermal conductivity detectors (TCD). The retention times and response factors were determined from cyclohexane, cyclohexene (4000 ppm in He, Linde), benzene (4000 ppm in He, Linde), and CO_2_ (10% vol in He, Linde) standards. The effluent stream was also qualitatively analyzed by sampling into the differentially pumped mass spectrometer chamber using an electronic needle control valve (Pfeiffer EVR 116), with the flowrate controlled by a regulator (Pfeiffer RVC 300) combined with a pressure gauge (Pfeiffer PKR 261) to keep a constant pressure set to 5.0 ×10^–6^ mbar in the mass spectrometer chamber. The mass spectrometer chamber was pumped by a turbo-molecular pump (Pfeiffer HiCube 80 Eco), which typically reached a background pressure of about 2×10^–8^ mbar. The mass spectrometer was operated in the continuous mass scanning mode (2 scans per minute) in the range from 10 to 100 m/z controlled by PV MassSpec software (Pfeiffer). Electron impact energy for the ionization was set to 70 eV. The sensitivity of the mass spectrometer for the desired molecules (cyclohexane, benzene, CO_2_, and O_2_) was determined using calibrated gas mixtures (certified analytical grade mixed gases, Messer, Air Products or Linde).

The oxidative dehydrogenation of cyclohexane was carried out under constant pre-set pressure (∼800 torr) maintained by a down-stream mass-flow controller (Brooks SLA5850) integrated into a regulation loop pumped by a diaphragm pump (Divac 1.4HV3), measured using a pressure transducer (Omega PX209), and controlled by a custom homemade software written in Python and at various temperatures (25–400 °C). All the conditions (temperature, partial pressures, and resident times) were adjusted in order to control the conversion below 2%. The catalyst powders were pretreated at 400 °C in 0.2 kPa O_2_ for 2 h before introducing the reaction conditions (SI, Section S3.1). Cyclohexene, benzene, and CO_2_ were only products detected in the MS and GC. Cyclohexene is a primary dehydrogenated product while benzene is a tertiary dehydrogenated product of cyclohexane. The secondary dehydrogenated product, cyclohexadiene, although thermodynamically favored,^33^ was not detected under the experimental conditions used in this work (*P*_cyclohexane_, *P*_O2_ = 0.01 to 0.2 kPa, 350 and 400 °C). The GO support was reduced under solvothermal conditions and tested for cyclohexane ODH reaction at 350 °C and has shown negligible activity.

The cyclohexane consumption rates were reported on a carbon basis as the sum of molar formation rates of cyclohexene, cyclohexane, and CO_2_ per g of CoFe_2_O_4_ (excluding the mass of RGO) per s (mol g_CoFe2O4_^–1^ s^–1^) while the molar formation rates of all products are reported as per g of catalyst (CoFe_2_O_4_ + RGO) per s (mol g_cat_^–1^ s^–1^). Selectivities to cyclohexene, benzene, and CO_2_ are reported on a molar basis (eq 1).
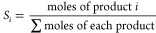
1

First-order deactivation rate constants (*k*_d_) are used to define catalyst stability during ODH:
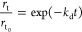
2where *r*_t_ and *r*_t_0__ denote, respectively, the total ODH rates at any time *t* and at initial contact with reactant (*t*_0_) and *k*_d_ is the deactivation constant (ks^–1^).

### 2.4 X-ray Photoelectron Spectroscopy (XPS)

The X-ray photoelectron spectra of the samples (before and after the ODH reaction) were measured using a modified ESCA 3 MkII multi-technique spectrometer (VG Scientific, East Grinstead, UK) equipped with a hemispherical electron analyzer operated in a fixed transmission mode to avoid overlap of Fe 2p and Co 2p spectra with, respectively, Co LMM and Fe LMM Auger spectra. The Mg Kα line was used as a source for the photoelectron spectra measurement. The binding energy scale of the spectrometer was calibrated using the Au 4f7/2 (84.0 eV) and Cu 2p3/2 (932.6 eV) photoemission lines. Binding energy steps for high-resolution scans were 0.1 eV with 200 ms dwell time per step. Measurements were performed at room temperature. The pressure in the XPS analysis chamber during spectra acquisition was 6 × 10^–9^ mbar. The samples were spread on an aluminum surface. The spectra were collected at a takeoff angle of 45° with respect to the macroscopic surface normal. High-resolution Fe 2p3/2, Co 2p3/2, O 1s, and C 1s photoelectron spectra were measured. O1s and C1s spectra are shown in the Supporting Information Section S2. The population of octahedral and tetrahedral sites was estimated from Fe 2p3/2 and Co 2p3/2 photoelectron spectra as described in the literature.^34−36^ The individual components of the deconvoluted spectra were described by a convolution of a Lorentzian function, representing the lifetime broadening, and a Gaussian function to account for the instrumental resolution. The Gaussian broadening was kept the same for different components. Shirley background^37^ was used to account for the inelastic scattering of photoelectrons in the high-resolution spectra. Spectra were calibrated for surface charging of samples using the method of internal standard and setting the major component of the C 1s peak to 284.6 eV.^38,39^ Quantification of the elemental concentrations was accomplished by correcting the photoelectron peak intensities for tabulated cross sections^40^ and the analyzer transmission function using CasaXPS software.^41^

### 2.5 X-ray Photoelectron Emission Microscopy (XPEEM)

Single-particle spectromicroscopy was carried out using X-ray photoemission electron microscopy at the Surface/Interface: Microscopy (SIM) beamline at the Swiss Light Source.^42^ XPEEM allows to probe many individual nanoparticles in a large ensembles simultaneously, giving insights into how the catalyst evolve as a function of temperature and reactants (See Supporting Information Section S3 for an XPEEM image). We investigated both small and large nanoparticle samples (7 and 12 nm, RGO-COFe1 and RGO-CoFe4) at 75 and 76 wt % loading on RGO, respectively, by drop casting these catalysts on a Si chip. To acquire X-ray absorption (XA) spectra, sequences of XPEEM images were recorded with photon energies ranging from 700 to 718 eV for the Fe L_3_ edge and 770 to 790 eV in the case of the Co L_3_ edge, as described earlier.^43^ For analysis, the images in each sequence are first corrected for drift, followed by the selection of the desired area on the support or the desired nanoparticle to obtain the XA spectra of individual nanoparticles or aggregates. The base pressure of the XPEEM chamber was around 5 × 10^–10^ mbar and around 2 × 10^–5^ mbar during the dosage of cyclohexane and O_2_ (around 1 × 10^–5^ mbar, each). The measurements were performed after gas exposure at various temperatures. XA spectra were recorded at room temperature, 250, 350, and 400 °C. The temperature was increased in steps of 2–5 °C per min. At each temperature, the reactant gases were dosed at a pressure of 2 × 10^–5^ mbar for 30 min, followed by evacuating the gases to achieve the pressure back to around 5 × 10^–10^ mbar and measuring the sample after the exposure to the reactants. In this way, both 12 and 7 nm samples were measured for around 1 h after each experimental condition. The measurement time included occasional re-alignment of the microscope, stabilization of thermal drifts of the sample, and obtaining the Co and Fe XA spectra.

### 2.6 Chemometric Analysis of XAS Data

The changes in XA spectra reflect the evolution of the concentration of components having distinct spectral profiles. The concentration and spectral profiles of these components were obtained by the Multivariate Curve Resolution by Alternate Least Square (MCR ALS) method^44−47^ using SpectroChemPy (a python based API).^44,46−50^ The XA spectra acquired from several particle aggregates (ca. 6) at each reaction condition were stored as a rectangular matrix “*X*” (*n* × *m* where *n* = number of spectra and *m* = number of photon energies (eV)) which was factorized into a *n* × *k* concentration matrix *C* of *k* components and a *k* × *m* pure spectra matrix *S*^t^ according to
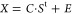
3where *E* is the matrix of the residual to be minimized.

The MCR ALS algorithm was initialized by providing initial guesses for the concentration matrix (*C*_0_) (using a non-negativity constraint) or spectral matrix (*S*^t^). The number of the initial guesses was chosen based on the observed intensities of the spectral features of the normalized experimental spectra (e.g., intensities at 779.3 and 780.3 eV from Co normalized spectra, vide infra, Section 3.4). As shown in the SI, similar results were obtained when initializing the algorithm using the computed spectra of the pure species from the literature. The initial guess for the spectral profile *S*_0_^t^ was then subjected to Principal Component Analysis (PCA) to identify the number of the spectral components (*S*^t^) and the corresponding concentration profiles (*C*) for each component at each reaction condition were calculated. These matrices (*S*^t^ and *C*) were then used for MCR ALS to re-construct the experimental spectral matrix *X̂*, minimizing the matrix of the residuals (*E*) using eq 3.

## 3 Results and Discussion

### 3.1 Catalyst Structure

**Figure 1:**
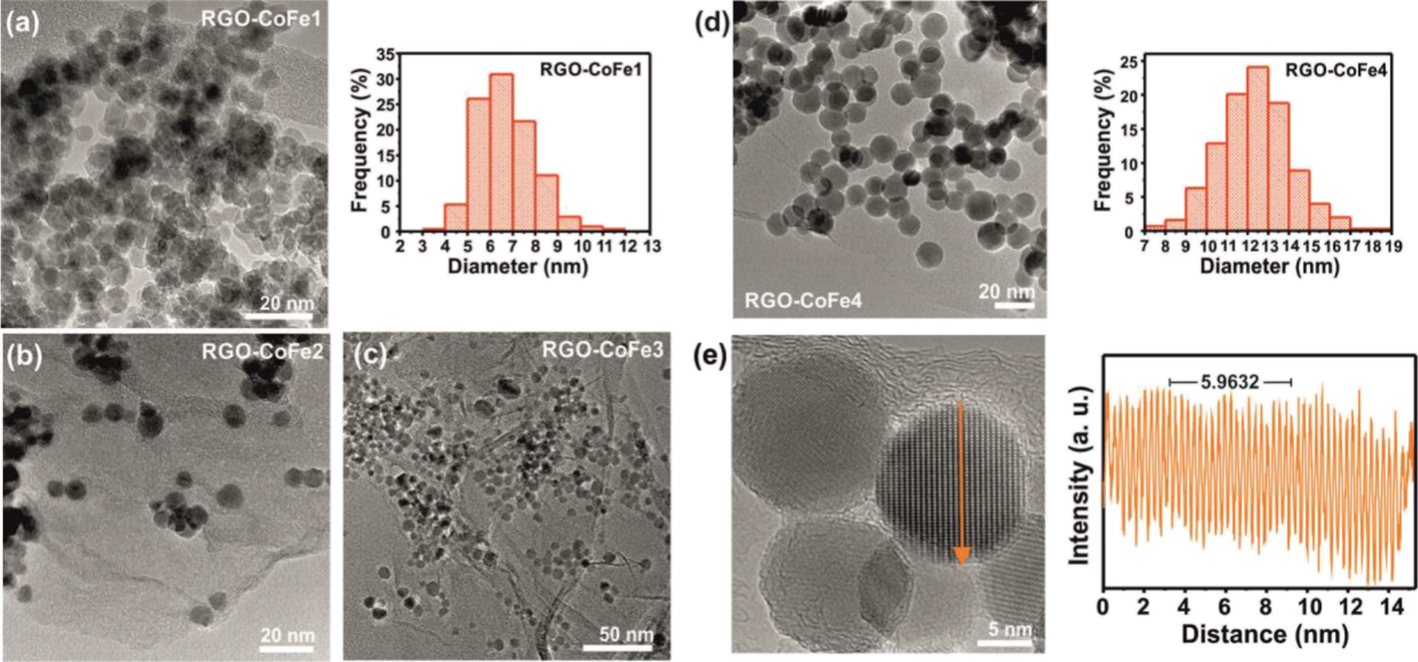
(a–c) TEM micrographs of composites obtained after functionalization of GO sheets with 7 nm (small) CoFe_2_O_4_ NPs. The nanohybrids were synthesized using three NPs/GO ratios, 1.66, 0.41, and 0.15, respectively. (d) CoFe_2_O_4_-RGO nanocomposite obtained after solvothermal treatment of GO in the presence of large (11.8 nm) CoFe_2_O_4_ NPs. The size distribution of small and large CoFe_2_O_4_ NPs after their attachment onto the GO support (RGO-CoFe1 and RGO-CoFe4, respectively) are included. (e) HRTEM micrograph of a crystalline nanosphere supported on RGO along with its corresponding intensity profile.

Figure 1a–c shows TEM micrographs of RGO-CoFe 1–3 nanohybrids with S-CoFe/GO ratios, 1.66, 0.41, and 0.15, respectively (See also Figure S2). The decreasing density of the number of CoFe particles on the GO support (per 50 nm^2^) with the decreasing CoFe/RGO ratio and the homogeneous particle distribution on the GO support was visually inspected. Figure 1d shows a micrograph of RGO-CoFe4 (CoFe/GO = 2.33) with L-CoFe NPs deposited on GO support following the synthesis protocol described in section 2.2. The size distribution of selected small (RGO-CoFe1, Figure 1a) and large (RGO-CoFe4, Figure 1b) CoFe_2_O_4_ NPs after the decoration of GO is shown next to the respective micrographs. In both cases, after measuring ca. 200 nanoparticles, Gaussian-like size distributions were observed (mean diameter = 6.7 ± 1.3 and 12.3 ± 1.7 nm, respectively) close to those obtained for the CoFe nanospheres without GO support (SI Section S1, Figure S1). For simplicity, the CoFe nanospheres (without GO support) from here onward will be referred as unsupported (S or L) CoFe NPs and the sizes of small NPs RGO-CoFe1–3 and big NPs RGO-CoFe4–5 will be referred to as 7 and 12 nm, respectively. The highly ordered character of the supported CoFe_2_O_4_ NPs (before the ODH reaction) was confirmed by HRTEM and their crystal structure was assessed. (Figure 1d). The interplanar distance of the crystal lattice measured from the intensity profile for the individual particle is in agreement with the lattice fringes of the [220] (*d* = 0.29 nm) of the inverse spinel cubic structure of CoFe_2_O_4_. The final loading (in wt %, Table 2) of CoFe_2_O_4_ NPs, attached to the GO surface and their thermal stability was assessed by TGA in the presence of O_2_ (SI Section S2, Figures S2 and S3) and presented in Table 2.

**Table 2:** Size Distribution and CoFe_2_O_4_ NPs Loading after the Decoration of the GO Support^a^

sample	NPs size (nm)	referred size (nm)	CoFe_2_O_4_ NPs contents (wt %, as determined by TGA)
S-CoFe	6.6 ± 1.4	7	
L-CoFe	11.8 ± 1.8	12	
RGO-CoFe2	6.9 ± 1.4	7	30
RGO-CoFe1	6.7 ± 1.3		75
RGO-CoFe3	7.1 ± 1.6		13
			
RGO-CoFe4	12.3 ± 1.7	12	76
RGO-CoFe5	12.4 ± 2		26

a Diameters of unsupported NPs (without GO) are included for comparison.

**Figure 2:**
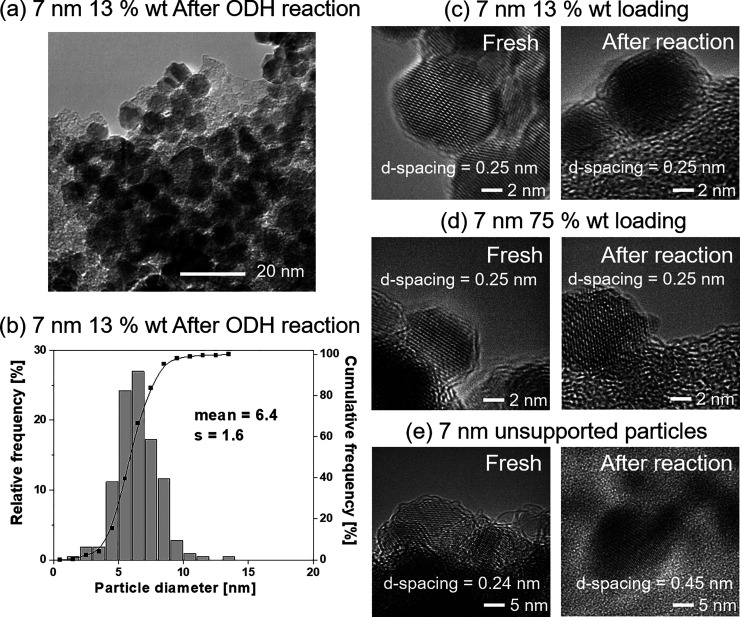
TEM image of the 7 nm 13 wt % RGO-CoFe1 particles after the cyclohexane ODH reaction (*T* = 350 °C, *P*_O2_ = *P*_cyclohexane_ = 0.2 kPa) (a) and corresponding particle size distribution (b). Panels (c), (d), and (e) show HRTEM images of 7 nm low loading (13 wt % RGO-CoFe3), high loading (75 wt % RGO-CoFe1), and unsupported particles, respectively, before and after the reaction.

The morphology of the catalysts was also analyzed before and after the ODH reaction (*T* = 350 °C, *P*_O2_ = *P*_cyclohexane_ = 0.2 kPa) to investigate any visual change in the particle shape and/or size as a function of reaction environment by TEM (Figure 2). No changes in the individual particle size due to agglomeration were observed, however, particle aggregates can be observed in the TEM images before (Figure 1a–d) as well as after (Figure 2a,b) the ODH reaction, especially for the high loading (75 wt %) sample. The particle size distribution also remained similar before and after the reaction. This is an indication that the oxygen functional groups present on RGO prevent possible agglomeration by holding the particles firmly to the support probably via strong sigma bonds (See TGA evidence in SI Section S2). After the reaction, the nanoparticles shape did not undergo significant variations, remaining mostly spheroid. Analysis of the HRTEM images (Figure 2c–e) clearly shows lattice fringes with a lattice spacing of about 0.25 nm, which agrees with the [311] plane of CoFe_2_O_4_ in agreement with the structure of the untreated NPs (before the ODH reaction).^51^The unsupported particles exposed lattice fringes of about 0.45 nm which corresponds to the 111 lattice plane or Fe_3_O_4_.^52^ This indicates a significant change in the particle structure such as the separation of phases into Fe_3_O_4_ and Co_3_O_4_^12^ and exposing Fe_3_O_4_ phase to the surface which was otherwise prevented when the particles were grafted on the RGO support.

In the next section, we examine the effect of GO support, wt % loading of CoFe particles on GO support, and particle size on cyclohexane ODH reaction rates and selectivities. We manipulate these parameters (particle size, support, and wt % loading) to control the selectivities toward cyclohexene and benzene.

### 3.2 Cyclohexane ODH Reactions on RGO-CoFe Nanohybrids: Role of Oxygen-Derived Species and the Effect of GO Support, wt % Loading, and Particle Size on ODH Reaction Selectivities

The ratio of void volume to catalysts mass in the reactor (∼0.98) and nondependence of cyclohexane consumption rates on the wt % loading (when normalized only by the total mass of CoFe_2_O_4_; vide infra) allowed to rule out homogeneous gas phase reactions under these conditions. The insulating nature of CoFe_2_O_4_ particles and linear increase of conversions with contact time (SI, Section S3.2, Figure S5) suggest that the heat loss time scales in our quartz-based reactor are of the similar order as the reactant residence times (1–30 s),^53^ allowing the resulting reactor behavior closely approaching isothermal behavior in such exothermic reaction environments, allowing to rule out heat and mass^54^ transfer corruptions in the measured rates. Thus, all reported reaction rates reflect closely the intrinsic dynamics of chemical events on the catalytic surfaces. The absence of cyclohexadiene product indicates its lower undetectable gas phase concentrations which might be quasi-equilibrated with its adsorbed species with faster subsequent C–H bond dissociation rate constants to produce the thermodynamically more stable product, benzene.

#### 3.2.1 Role of Oxygen-Derived Species in Reaction Rates and Selectivities

**Figure 3:**
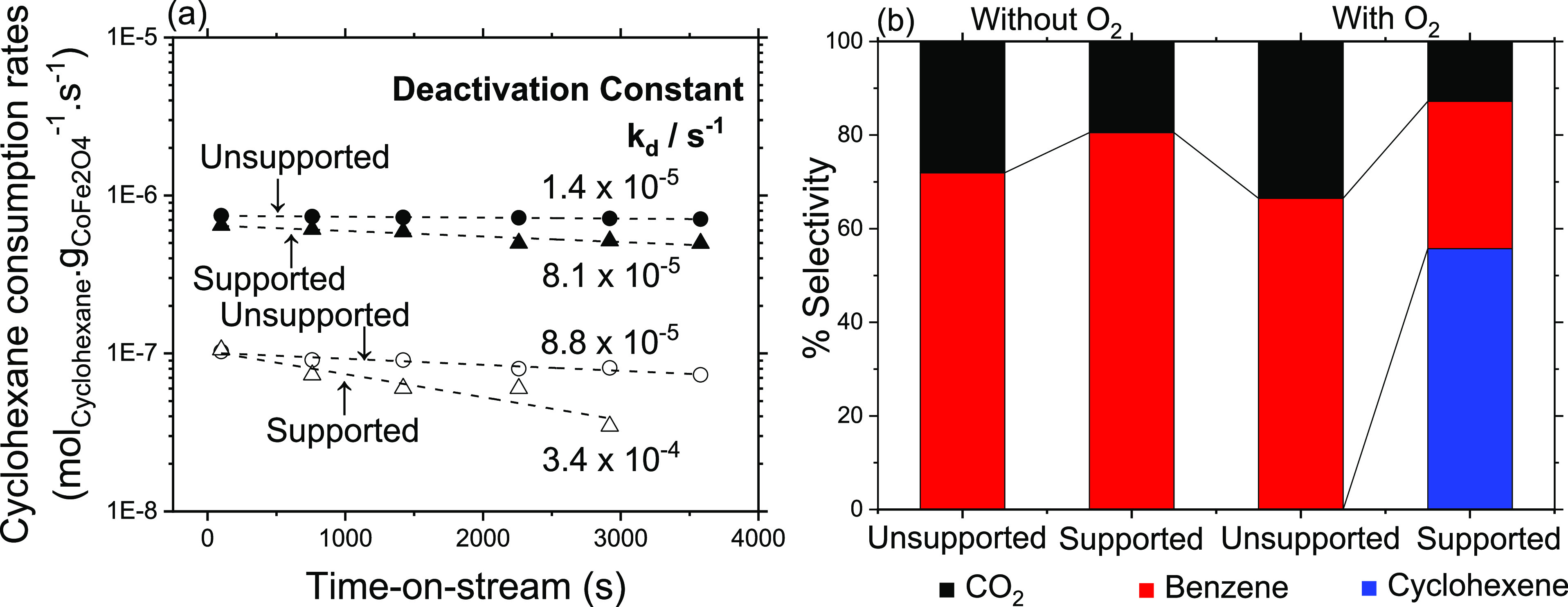
(a) Cyclohexane consumption rates (per g of CoFe_2_O_4_ (excluding RGO) per s) on 12 nm unsupported (circles, L-CoFe) and supported (triangles, 26 wt % on RGO-CoFe5) CoFe_2_O_4_ particles, with O_2_ (*P*_cyclohexane_ = *P*_O2_ = 0.2 kPa) in the reactant feed (filled symbols) and without O_2_ (*P*_cyclohexane_ = 0.2 kPa) (empty symbols) at 350 °C. The dotted lines are the best fits to eq 2. (b) Corresponding selectivities measured under similar conversion (∼0.25%).

Figure 3a shows cyclohexane consumption rates (normalized by only the mass of CoFe_2_O_4_) as a function of time-on-stream at 350 °C measured on 12 nm unsupported (circles, L-CoFe) and supported (triangles, 26 wt % on RGO, RGO-CoFe-5) CoFe_2_O_4_ particles, with O_2_ (*P*_O2_ = 0.2 kPa) and cyclohexane (*P*_cyclohexane_ = 0.2 kPa) in the reactant inlet feed (filled symbols) and without O_2_ (nonoxygenated conditions, *P*_cyclohexane_ = 0.2 kPa) (empty symbols). Figure 3b shows corresponding selectivities toward cyclohexene, benzene, and CO_2_, measured under similar cyclohexane conversion levels (∼0.25%). The slopes of trends in Figure 3a give the first-order deactivation constant (−*k*_d_, eq 2) which is an indication of the mean catalyst life (*k*_d_^–1^, s). The higher value of the deactivation constant on RGO-supported CoFe_2_O_4_ particles under nonoxygenated conditions (3.4 × 10^–4^ s^–1^) indicates the combination of the prevalent deposition of cyclohexane-derived species on active sites and formation of oxygen vacancies. Under similar nonoxygenated conditions, the unsupported particles show much more stable total ODH rates apparently because of the total combustion of adsorbed cyclohexane-derived species much more effectively as evident from the larger selectivity toward CO_2_ compared to supported particles.

**Figure 4:**
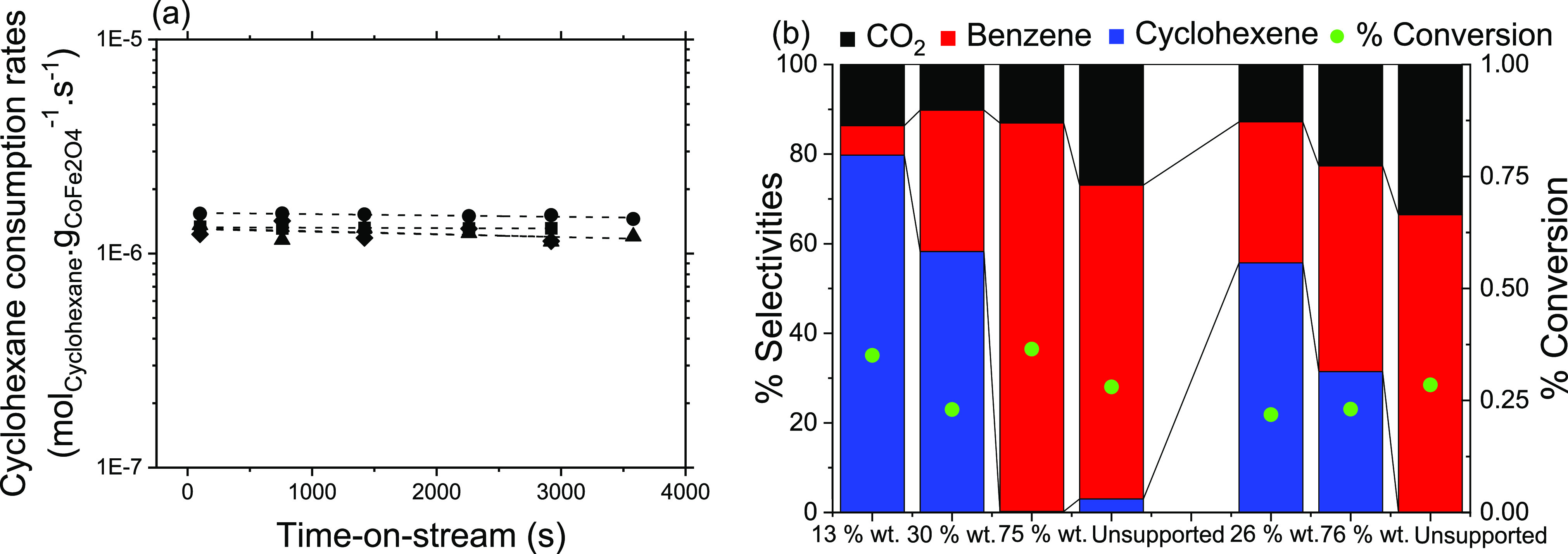
(a) Cyclohexane consumption rates as a function of time-on-stream (s) measured on small (7 nm) (filled symbols) CoFe_2_O_4_ particles deposited on RGO with various amounts (in wt % )(Unsupported 7 nm particles (S-CoFe, ●),75 wt % (RGO-CoFe1, ■), 30 wt % (RGO-CoFe2, ◆), 13 wt % (RGO-CoFe3, ▲)) at 350 °C and *P*_cyclohexane_ = *P*_O2_ = 0.2 kPa. The dotted lines are the best fits to the eq 2. (b) Corresponding selectivities toward CO_2_ (black), benzene (red), and cyclohexene (blue) and % cyclohexane conversion (green dots) on 7 nm samples (13, 30 and 75 wt % and unsupported) and 12 nm samples (26, 76 wt % and unsupported)

The initial cyclohexane consumption rates are almost an order of magnitude higher under oxygenated conditions (*P*_cyclohexane_ = *P*_O2_ = 0.2 kPa) and exhibit higher stability for supported particles than when measured under nonoxygenated conditions. The significant differences in the initial rates (extrapolated to 0 s) between oxygenated and non-oxygenated conditions strongly indicate the formation and involvement of oxygen-derived species in C–H bond dissociation and also suggest that the lattice O-atoms are less reactive toward C–H bond abstraction. Furthermore, almost similar rates were observed for unsupported and supported catalysts (Figures 3a and 4a) and in all wt % loadings (Figure 4a, 7 nm) in oxygenated conditions confirm the sole kinetic relevance of these oxygen species in C–H bond abstraction. Such oxygen-derived species could involve dioxygen cation (O_2_^+^), superoxide ion (O_2_^–^), peroxide ion (O_2_^2–^),^55^ and monoatomic adoxygen species^11,30,55,56^ formed by activation of a strong O-O bond. The existence of monoatomic adoxygen species has been recently proposed for spinel oxides of cobalt such as CoFe_2_O_4_^30^ and Co_3_O_4_.^11,57^ On CoFe_2_O_4_, O_2_ can be activated either on Fe or Co cation pair to form reactive adoxygen species (O*) with coordinative interaction between the O adatoms and the d orbital of the metal centers (Fe and Co). The higher initial total ODH rates under oxygenated conditions suggests that these newly formed O* species might activate C–H bond via H-abstraction much more efficiently than the lattice O-atoms.

The selectivities, on the other hand, are significantly different for these samples under oxygenated conditions (Figure 3b). The higher selectivity toward CO_2_ observed on unsupported particles in oxygenated conditions and no detection of cyclohexene reflect that the cyclohexane derived species must remain strongly bound and efficiently undergo C–C bond cleavage and O-insertion reactions to form CO_2_ or undergo subsequent C–H bond cleavage to form full dehydrogenated product benzene. The RGO supported particles, however, showed a remarkably high selectivity toward cyclohexene (56%, Figure 3b) under oxygenated conditions with significantly lower selectivity toward CO_2_ and benzene might indicate that the cyclohexane-derived species are less strongly bound on these O* species and desorb after primary dehydrogenation before undergoing subsequent dehydrogenation reaction. This observation is consistent for all the samples investigated in this work. The selectivity toward cyclohexene (Figure 4b) increase with decreasing the wt % content of CoFe_2_O_4_ particles on the RGO support for both particle sizes under approximately similar conversion levels, while the initial cyclohexane consumption rates remained similar and stable for a given size for all wt % loadings. Such significant differences in the selectivities as a function of wt % content of CoFe_2_O_4_ on RGO without compromising the cyclohexane consumption rates requires mechanistic understanding relating identification of the active sites and their respective contributions to cyclohexane selectivities. What follows next is the discussion to understand the reasons for different selectivities observed on these samples as a function of different wt % contents of CoFe_2_O_4_ NPs on RGO.

#### 3.2.2 Coverage, Stability, and Reactivity of O* Species

The differences in the selectivities observed in Figure 4b with various wt % CoFe contents might reflect the differences in the O* coverage on the catalyst surface. The O* coverage can vary as we increase the partial pressures of O_2_ or wt % contents of CoFe particles keeping the O_2_ pressures constant. To investigate this fact, we measured the selectivities on the RGO-CoFe1 (7 nm 75 wt %) sample as a function of varying O_2_ partial pressures (O_2_/cyclohexane ratios 1:1 to 25:1, Section S3.3, Figure S6). Surprisingly, the selectivities toward cyclohexene (∼2 to 5%) and benzene (75–78%) were found to be insensitive to O_2_/Cyclohexane ratios (in the range of 1–25) (See Figure S6). Such insensitivity and low cyclohexene selectivity can no longer be explained by different kinetic regimes created by varying O_2_/Cyclohexane ratios which would certainly lead to high relative O* coverage at a higher O_2_/cyclohexane ratio.

This prompts us to investigate the reactivity or stability of the O* species as it can significantly affect selectivities irrespective of its coverage. The highly active O* species can be rapidly consumed by cyclohexane-derived species to form benzene through complete dehydrogenation reactions in a single sojourn. The corollary of this statement would be the less strongly the O* are bound to the surface more effective they are in H-abstraction.^11,56^ This also reflects the reversibility of O_2_ activation during cyclohexane ODH reactions and the effect of RGO support on the formation and stability of these O* species. Therefore, we probe the stability and reactivity of O* species by comparing the magnitude and reaction orders for the net O_2_ consumption rates during cyclohexane ODH reactions.^58^ In general, the chemical equation for the reaction between O_2_ and cyclohexane (R) leads to CO_2_, H_2_O, and other O-containing products P:
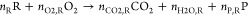
4where *n_i_* is the stoichiometric coefficient for species *i*.

The net rate of oxygen consumption (*r*_O_2_, R_) and the net rate of cyclohexane consumption (*r*_R_) are related via the reaction stoichiometries *n*_R_ and *n*_O2, R_ as follows:
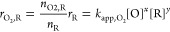
5where *k*_app, O_2__is the apparent rate constant for O_2_ consumption and [O] and [R] are the pressures of O_2_ and cyclohexane raised to their respective reaction orders (*x* and *y*), respectively.

Table 3 summarizes the apparent reaction order for O_2_ for 7 and 12 nm CoFe_2_O_4_ particles on RGO with various wt % loadings and respective range of oxygen consumption rates measured at 400 °C and constant cyclohexane partial pressure (*P*_cyclohexane_ = 0.2 kPa). The oxygen consumption rates differ by two orders of magnitude (0.35–56 mol_O2_ mol_CoFe2O4_^–1^ s^–1^) on 7 nm samples by varying the wt % content on RGO from 13 to 75 wt %, while for 12 nm, *r*_O_2_, R_ varies by an order of magnitude from 25 to 75 wt %. The largest O_2_ consumption rates measured on 7 and 12 nm particles are on 75 wt % samples, suggesting rapid O* scavenging from the surface. This rapid O* scavenging may result in lowering the O* coverages to below equilibrium, resulting in the apparent reaction orders with respect to O_2_ for both these samples 0.75 and 0.59, respectively.

**Table 3:** Oxygen Consumption Rates and Kinetic Dependencies during Cyclohexane ODH Reactions on CoFe_2_O_4_ Particles (7 and 12 nm) with Varying Amounts of Loadings on RGO (in wt %) Obtained from Figure 5

	apparent reaction order of O_2_	O_2_ consumption rates (mol_O2_ mol_CoFe2O4_^–1^ s^–1^)
7 nm		
13 wt %	–0.052 ± 0.040	0.35–0.4
30 wt %	0.20 ± 0.07	1.22–1.84
75 wt %	0.75 ± 0.37	48–56
12 nm		
26 wt %	0.098 ± 0.040	0.2–0.3
76 wt %	0.59 ± 0.02	0.8–3.6

The lower relative magnitudes of O_2_ consumption rates along with the smaller corresponding reaction orders observed on samples with lower CoFe_2_O_4_ wt % contents (30, 26, and 13 wt %) may suggest either one of

(i) The O* species formed on these samples are only moderately active and therefore are not scavenged as quickly as observed on higher wt % samples (75 wt %) under similar O_2_ partial pressures. Thus, on lower wt % samples, O_2_(g) may dissociate and then recombine much more rapidly than the rate of O* scavenging by cyclohexane-derived species.
(ii) The cyclohexane-derived intermediates remain less strongly bound on these smaller wt % samples, thus consuming small amount of O* species less rapidly than the higher wt % samples.

These dependencies on O_2_ pressures, along with the insensitivity of selectivities on O_2_–cyclohexane ratios (Figure S6), and significant differences in the initial ODH rates with and without O_2_ (Figure 4a) validate the fact that the CoFe_2_O_4_ surfaces mostly remain covered predominantly by O* species and O_2_ activation is kinetically irrelevant (fast) on these catalysts. We further speculate that such a fast O_2_ dissociation into O* species and their retransformation into molecular oxygen may reflect the nature of the O* species to be monoatomic oxygen. This is because such splitting of O_2_ into monoatomic O-adatoms and their retransformation into molecular O_2_ probably does not require any additional reversible charge transfer processes (e.g., electron transfer from Fe^3+^ to O_2_ to form O_2_^2–^ or O_2_^–^ species as reported by Rushiti and Hattig^30^) which might be kinetically limited. This in turn would be reflected in either shift in selectivities from benzene to cyclohexene with increasing O_2_–cyclohexane ratios (Figure S6) or a linear dependence of oxygen consumption rates on O_2_ pressures (linear at low O_2_ pressures and nonlinear at high O_2_ pressures, etc.) on all the samples investigated due to change in the surface coverage of these anionic species, which is not observed here. This speculation is supported by previous experimental and theoretical studies,^59^ however, requires further investigation using time-resolved transient methods which allow following fast O_2_ activation processes on spinel oxides.

The higher apparent reaction order with respect to O_2_ observed on 75 wt % loading samples can be then explained by the following: (ii) the cyclohexane-derived species can remain strongly bound to the surface, leading to consecutive H-abstraction and therefore the faster effective scavenging of O* species. This would result in a decrease in the O* coverage to below equilibrium, and as a result, the O_2_ activation step becomes irreversible, causing the corresponding reaction order to approach unity. On the lower wt % samples, the cyclohexane-derived species are less strongly bound and therefore consuming O* species less rapidly and the surface would still remain prevalently covered with O* species causing corresponding reaction orders close to zero.

**Figure 5:**
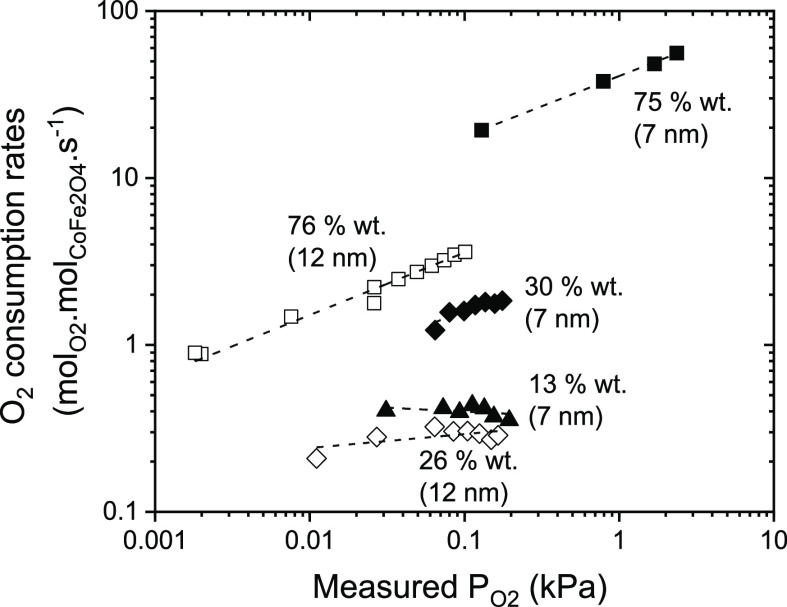
Dependence of oxygen consumption rates during cyclohexane ODH reactions on O_2_ pressures, measured on 7 nm CoFe_2_O_4_ particles supported on RGO with loadings of 75 wt % (RGO-CoFe1, ■), 30 wt % (RGO-CoFe2, ◆), and 13 wt % (RGO-CoFe3, ▲) and on 12 nm CoFe_2_O_4_ NPs supported on RGO with loadings 76 wt % (RGO-CoFe-4, □), 26 wt % (RGO-CoFe-5, ◊) at 400 °C and *P*_cyclohexane_ = 0.2 kPa.

This fact indicates that there may be two types of active sites present on the CoFe NPs surface that can either bind cyclohexane derived moieties strongly or weakly and their relative abundance depends on the wt % content of NPs on RGO support. Figure S7 shows the correlations between benzene, CO_2_, and cyclohexene formation rates and O_2_ consumption rates further suggest that the cyclohexane-derived species can remain strongly bound on the surface and subsequently consume O* species to form benzene at higher wt % loading samples. We further observe that the 7 nm 30 wt % sample showed a higher apparent reaction order (0.2) and O_2_ consumption rates compared to 12 nm sample with almost similar wt % loading (26 wt %) (Figure 5). This again prompts that these smaller nanoparticles (7 nm) exhibit higher abundance of these sites with stronger affinity toward cyclohexane than on the 12 nm particles at similar wt % loading.

#### 3.2.3 Indication of the Presence of Two Types of Active Sites with Different Affinity toward Cyclohexane-Derived Intermediates and Controlling Their Abundance

In this section, we support the presence of two different types of active sites with different affinities toward cyclohexane-derived species by observing benzene and cyclohexene formation rates on different wt % samples and their dependence on cyclohexane pressures. The relative affinity of these sites toward cyclohexane-derived species (producing benzene and cyclohexene accordingly) and their abundance on the surface of the NPs would be evident through such pressure-dependent experiments. We show how the relative abundance of these sites depends on the wt % loading and how it affects the benzene and cyclohexene formation rates and how it can be precisely tuned.

**Figure 6:**
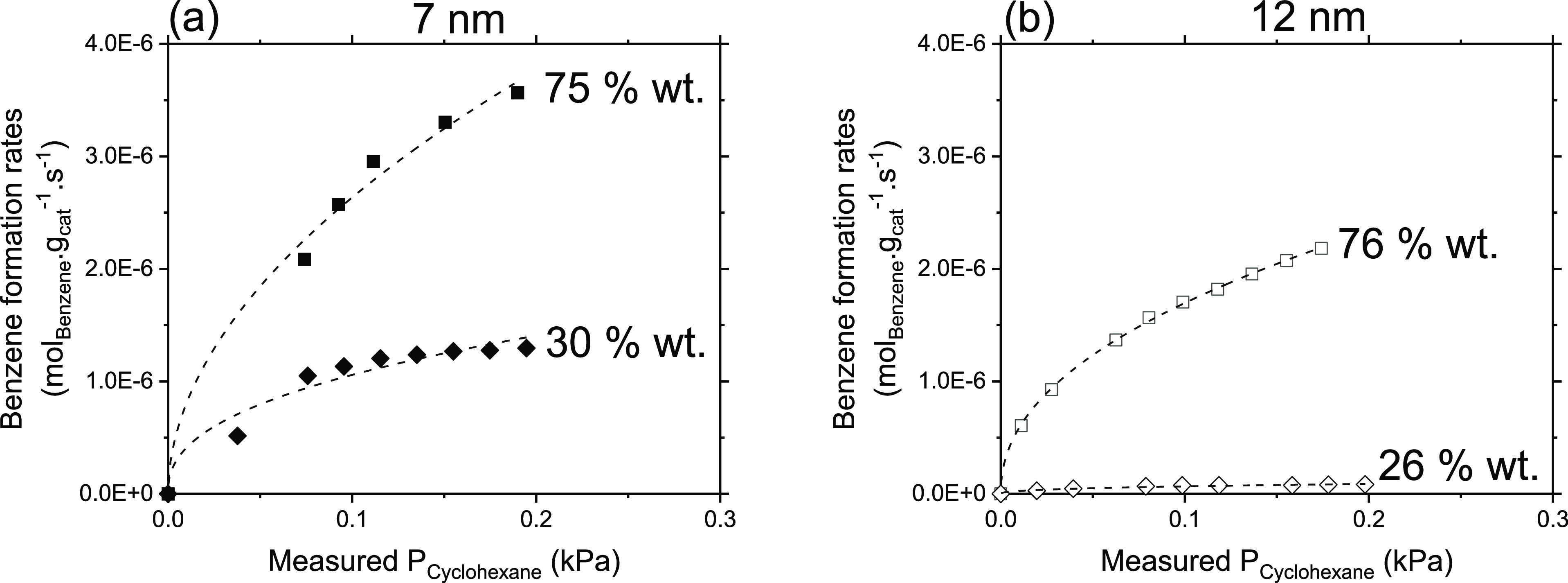
Benzene site-time yields (per g of catalyst (CoFe_2_O_4_ + RGO) on (a) 7 nm samples with 75 wt % (■), 30 wt % (◆) loadings on RGO (No benzene was detected on 13 wt % 7 nm sample) and (b) 12 nm samples with 76 wt % (□), 26 wt % (◊) loadings on RGO at 400 °C and *P*_O2_ = 0.2 kPa. The dashed lines are the guide to the eye.

**Figure 7:**
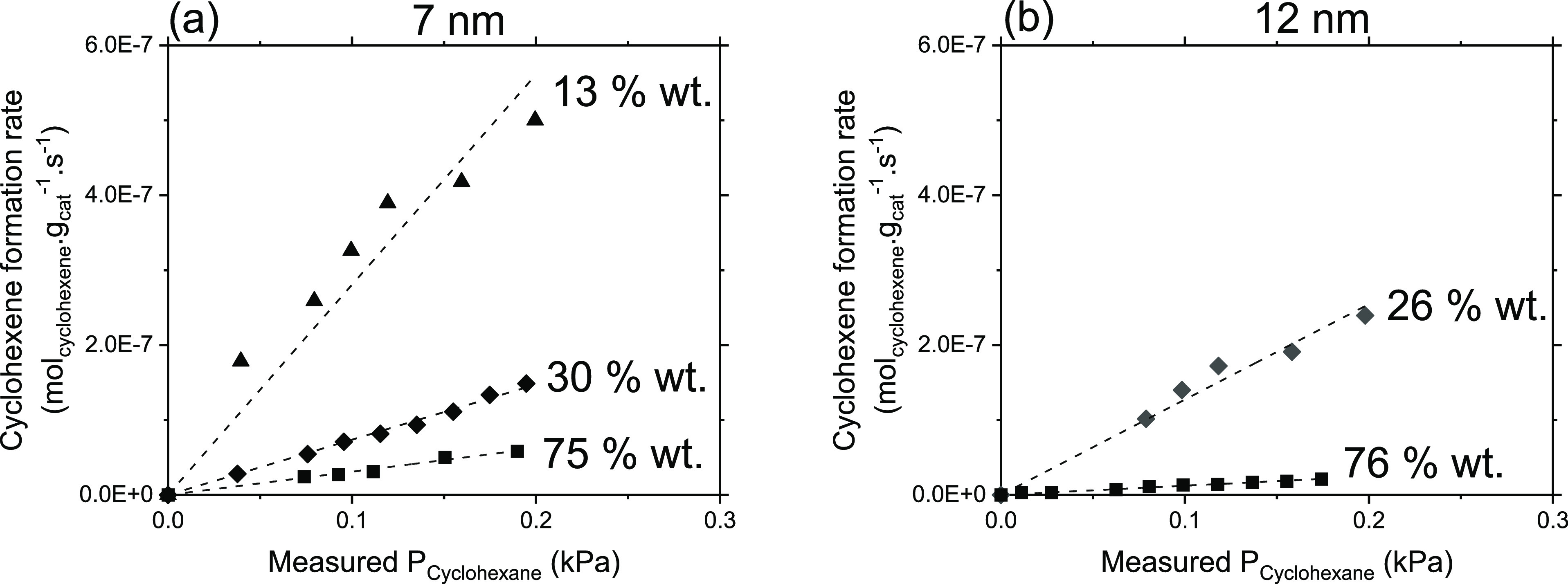
Cyclohexene site-time yields (per g of catalyst (CoFe_2_O_4_ + RGO) on (a) 7 nm samples with 75 wt % (■), 30 wt % (◆), and 13 wt % (▲) loadings on RGO and (b) 12 nm samples with 76 wt % (□), 26 wt % (◊) loadings on RGO at 400 °C and *P*_O2_ = 0.2 kPa. The dashed lines are the best fits to eq S11.

Figure 6a,b shows benzene formation rates measured on 7 and 12 nm particles deposited on RGO, respectively, as a function of cyclohexane pressures measured at the outlet of the reactor. These curves become quickly insensitive to the cyclohexane pressure only on lower wt % samples (30 and 26 wt %) while they show a clear asymptotic behavior (linear at lower and nonlinear at higher cyclohexane pressures, respectively) on higher wt % loadings (75 wt %), in line with the O_2_ pressure dependencies observed in Figure 5. At variance, the cyclohexene formation rates (Figure 7a,b) remain linear for all the wt % loadings under similar cyclohexane pressures. These differences in the dependence of the yields of benzene and cyclohexene on similar cyclohexane pressures suggest that the surfaces of these NPs may exhibit two types of active sites (Site A and Site B).

The linear dependence of cyclohexene formation rates upon cyclohexane pressures indicates that the active sites on which this partial dehydrogenation occurs are prevalently unoccupied, but their relative abundance decrease with increasing wt % contents of CoFe NPs, as evident by decrease in cyclohexene rates with increasing wt % contents observed on both NPs sizes (Figure 7a,b). Since these sites perform only partial dehydrogenation, they must therefore possess weaker affinity toward cyclohexane and derived species. Considering the catalyst surface fully covered by O* species (as evident in Figure 3, Figure 5, and linear nature of curves in Figure 7), the cyclohexene formation could be catalyzed by O* species that remain prevalently vacant while assuming C–H bond dissociation on O* species as a rate-limiting step in all the samples, allowing us to assign sites B as O* species that form preferentially cyclohexene. Such C–H bond cleavage could probably occur via homolytic cleavage on the surface that is predominantly covered by adjacent O* species (O-adatoms), as previously shown for methane activation^56^ and cyclohexane oxidative dehydrogenation on Co_3_O_4._^11^

The insensitive nature of benzene formation rates on cyclohexane pressures observed on low loading samples (30 and 26 wt %) and nonmonotonic behavior on high wt % content samples additionally suggest that the active sites (Site A) which conduct full cyclohexane dehydrogenation in a single surface visit (vide infra, Section 3.5) are mostly occupied by cyclohexane and derived intermediates. Note that despite the fact that the cyclohexane pressures in both Figures 6 and 7 are similar, still one type of sites remains largely unoccupied (Figure 7, Site B, O* species) whereas the types of sites are almost fully covered. Moreover, we see that the lower wt % samples (30 wt % in 7 nm and 26 wt % in 12 nm) show almost independence of benzene formation rates on cyclohexane pressures, meaning that the active sites on the lower wt % samples are almost fully covered (saturated) by cyclohexane and derived moieties than on high wt % loading samples, where some of these sites are still unoccupied. These data clearly suggest that the active sites which conduct full dehydrogenation, hence possess stronger affinity toward cyclohexane and derived species, are also present on the surface of the NPs, and their abundance depend on the wt % contents of CoFe NPs on RGO. The asymptotic nature and high magnitudes of benzene formation rates on high wt % samples and the corresponding linear dependence of cyclohexene formation rates with lower magnitudes allow us to conclude that the high wt % sample possesses site A in higher abundance, which strongly bind cyclohexane derived species and convert them to benzene via subsequent C–H bond abstraction. In doing so, the O* species are rapidly consumed below their equilibrium coverage, as observed in Figure 5, resulting in a decrease in their abundance causing lower cyclohexene formation rates, as observed in Figure 7.

Thus, we used cyclohexene and benzene as markers to identify the existence of two sites on the surfaces of the NPs (Site A and Site B): one site performs partial dehydrogenation, hence possesses weaker affinity toward cyclohexane and intermediates and remain largely unoccupied (Site B, O* species), while the other type performs full dehydrogenation, hence possesses strong affinity toward cyclohexane and intermediates and is almost fully saturated with cyclohexane and derived species (Site A). The relative abundances of these sites depend on the wt % contents of CoFe NPs on RGO. As we increase the wt % contents, we have a clear indication that we increase the abundance of Site A on the NPs surface, resulting in higher rates of benzene formation with nonlinear dependence on cyclohexane pressures. The increased abundance of Site A eventually leads to a decrease in O* coverage below equilibrium, as observed in Figure 5 (causing linear dependence or *r*_O2,R_ on O_2_ partial pressures), hence leading to a decrease in cyclohexene formation rates with increasing wt % contents (Figure 7a,b).

As discussed in Section 3.2.2, the nature of Site B appears to be reactive O* species; however, further characterization of this kinetically fast step using time-resolved transient methods is necessary to identify the nature of O* species (O-adatoms, O_2_^2–^, etc.) and follow how exactly O_2_ activation occurs on spinel oxides. To identify the nature of site A, we investigate these catalysts using XPS and XA spectroscopy in the next sections.

### 3.3 Analysis of Site A Using XPS

**Figure 8:**
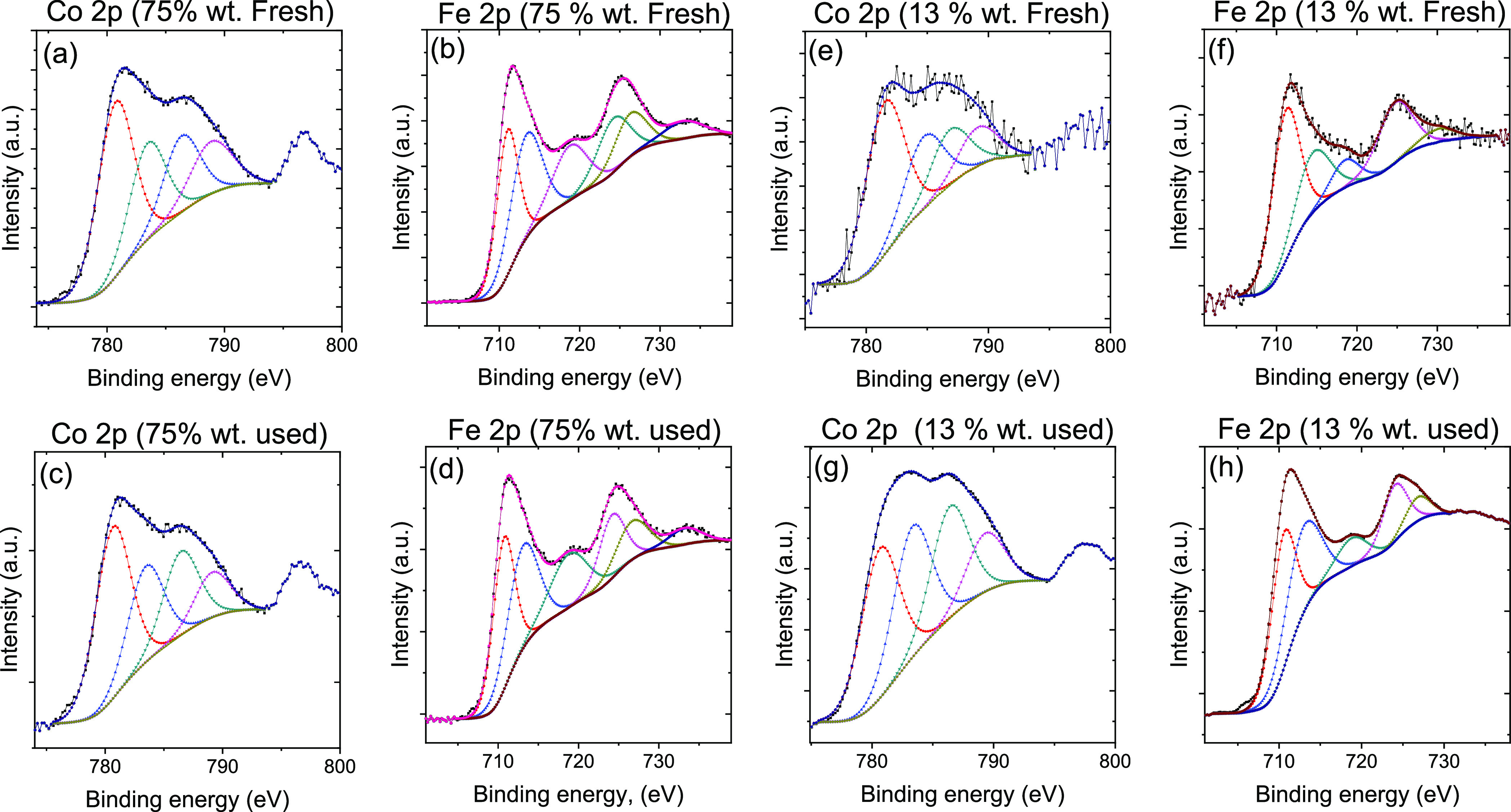
XPS high-resolution spectra of Co 2p and Fe 2p of the 7 nm 75% (RGO-CoFe1) and 13 wt % (RGO-CoFe3) loading CoFe_2_O_4_ as-synthesized (fresh) and after reaction (used) catalysts powders (reaction conditions: 350 °C, *P*_O2_ = 0.2 kPa, *P*_cyclohexane_ = 0.2 kPa). Corresponding C 1s and O 1s spectra are in Figure S9.

The XPS analysis was performed to understand how the wt % content of CoFe_2_O_4_ nanoparticles on the RGO support would affect the catalysts surface structure as a function of reaction conditions to explain the activity/selectivity observed above in Section 3.2. We investigated two samples with a similar particle size (7 nm) with high wt % (75%, RGO-CoFe-1) and low wt % contents (13 wt % RGO-CoFe-3) before (fresh) and after (used) the ODH reaction (*T* = 350 °C, *P*_O2_ = *P*_cyclohexane_ = 0.2 kPa). Figure 8 shows core-level spectra of Co 2p3/2 and Fe 2p3/2 photoelectrons and the composition of analyzed samples calculated from integrated intensities of spectra including intensities of C 1s and O 1s spectra and assuming homogeneous samples are summarized in Table 4. The probing depth is determined by the inelastic mean free path of photoelectrons in cobalt ferrite which amounts ∼1.3 nm as calculated from the TPP 2 M equation.^60^

**Table 4:** Composition (in %) of Superficial Layers on 7 nm Catalyst samples

sample	Co	Fe	C	O
75 wt % -RGO-CoFe1 (fresh)	2.9	4.3	57.0	35.8
75 wt % -RGO-CoFe1 (used)	3.0	7.0	52.0	38.0
13 wt % -RGO-CoFe3 (fresh)	0.5	1.0	72.2	26.3
13 wt % -RGO-CoFe3 (used)	0.8	1.4	61.4	36.4

It should be also mentioned that the accuracy of the quantitative analysis depends on the choice of background which is rather complicated in the case of spectra of Fe 2p and Co 2p photoelectrons (for a more detailed discussion, see previous study.^61^) The surface concentration of iron as well as cobalt in fresh samples is lower than that in samples after the catalytic reaction while the concentration of carbon was higher for the fresh samples and lower for the used samples, indicating that the NPs surfaces in the fresh samples are most probably covered by RGO that is partially removed during the catalytic reaction. Such covering of the nanoparticle surface by RGO could result in masking of active sites, thus decreasing their relative population on the surface of the nanoparticles. Some of these sites can be recovered but only partially during the reaction due to the partial removal of RGO.

The high-resolution Co 2p3/2 spectra for all the samples (before and after the reaction) are deconvoluted into 4 components (Figure 8). The component at 781 eV along with its shake-up satellite at 786.5 indicate Co^2+^ cations at the octahedral site. The peak at 783.5 eV is attributed to Co^2+^ cations at the tetrahedral site as also indicated by the corresponding satellite peak at 789.1 eV. The structure of the spectra of Co 2p3/2 photoelectrons that is consistent with the oxidation state of cobalt is +2 in all measured samples.^36^

The high-resolution spectra of Fe 2p photoelectrons are presented in Figure 8 and are characteristic of iron in oxidation state +3.^36,62,63^ The Fe 2p_3/2_ spectra are composed of three peaks located at 710.8, 713.5, and 718.8 eV and ascribed to Fe^3+^ cations at the O_h_ sites, Fe^3+^ cations at T_d_ sites and a satellite feature, respectively. The deconvoluted spectra do not indicate the presence of a component assignable to Fe^2+^. The Fe 2p_1/2_ spectra can also be decomposed into three peaks at 725, 727, and 733.5 eV and ascribed to Fe^3+^ species at O_h_, T_d_, and satellite feature, respectively.

**Figure 9:**
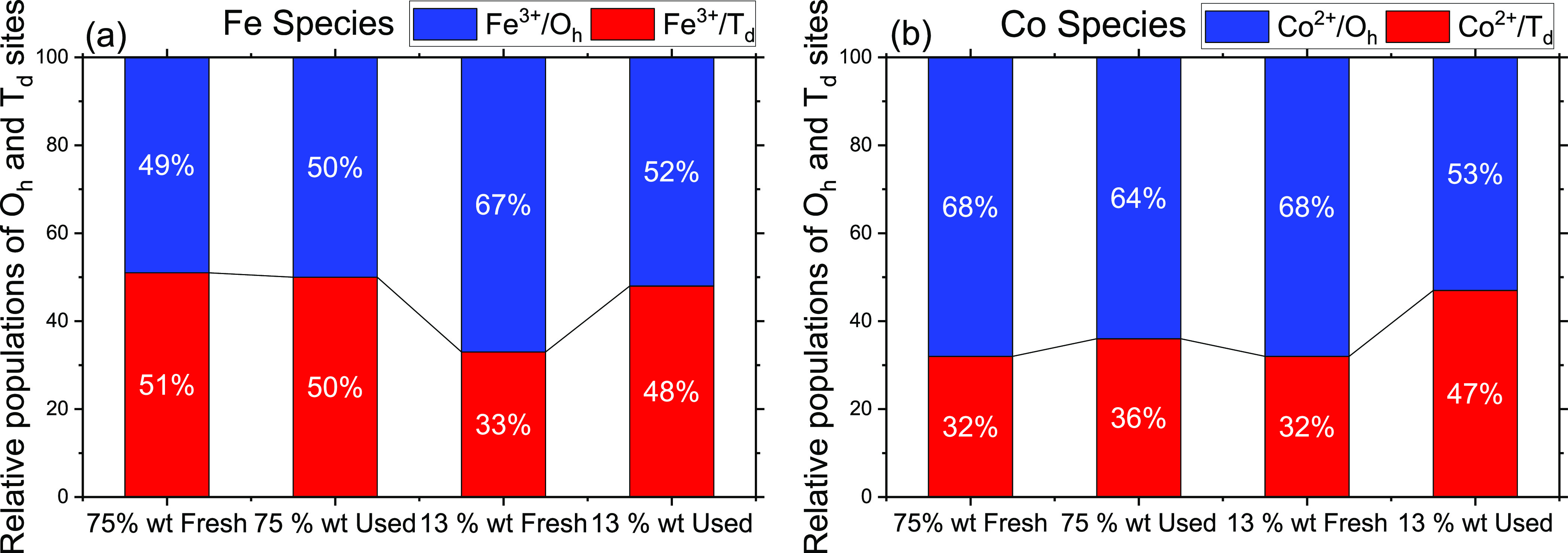
Population of octahedral and tetrahedral sites occupied by (a) Fe and (b) Co in the as-synthesized (Fresh) and after ODH reaction (used) 7 nm CoFe_2_O_4_ catalysts in 75% (RGO-CoFe1) and 13 wt % (RGO-CoFe-3) samples. The population of O_h_ and T_d_ species was determined from the respective peak areas shown in Figure 8. ODH reaction conditions for used catalysts are 350 °C, *P*_O2_ = 0.2 kPa, *P*_cyclohexane_ = 0.2 kPa).

The population of octahedral and tetrahedral sites on the particle surface was determined from deconvoluted high-resolution spectra of Co 2p3/2 and Fe 2p3/2 photoelectrons and are summarized in Figure 9a,b. The peak area is used to quantify the fraction of the total amount of Co and Fe in a unit cell occupying O_h_ and T_d_ sites in the fresh as well spent samples in both the loadings represented in Figure 9 in the form of bar graphs.

Figure 9b shows that the fresh 75 and fresh 13 wt % samples exhibit similar relative populations of surface Co^2+^ cations at O_h_ and T_d_ coordinations; however, the relative populations of Fe^3+^ cations at these coordinations are significantly different (Figure 9a). The population of Fe at tetrahedral sites is higher in 75 wt % fresh sample compared to those in 13 wt % fresh sample. Furthermore, unlike 13 wt %, the 75 wt % sample did not undergo any significant changes in terms of the relative population of Co and Fe octahedral and tetrahedral species. We propose and later support the hypothesis that these Fe^3+^/T_d_ species which are abundant on the surface of the 75 wt % sample, bind cyclohexane derived moieties strongly and are responsible for the complete cyclohexane dehydrogenation to benzene as observed in Figure 4b.

As observed in the TEM images (Figure 1c) in the 13 wt % fresh sample, the nanoparticles are well dispersed and are less crowded with direct contact with the RGO support. In such case, as suggested above, the RGO support could cover a fraction of the surface of the nanoparticles. The lower relative population of the Fe^3+^/T_d_ species in 13 wt % fresh sample compared to 75 wt % fresh sample and the fact that RGO could cover the fraction of the particle surface allow us to postulate that these Fe^3+^/T_d_ species could interact with the RGO support during the synthesis and thus can be masked. At high temperature (350 °C) during the reaction, some of the RGO layer could be removed due to the possible combustion resulting in exposing these Fe^3+^/T_d_ species, thus slightly increasing their relative population (15%) in the used 13 wt % used sample (Figure 9a). A similar increase in the Co^2+^ tetrahedral species, however, could be due to their migration from octahedral to tetrahedral locations as a response to reaction temperature and/or presence of reactants as suggested by XAS data in the next section. We show that these qualitative observations are consistent with the semi-quantitative XAS observations, suggesting that Fe^3+^/T_d_ could be possibly masked by the RGO support, especially in the 7 nm particles and unavailable for the coordination exchange with Co^2+^ cations during the reaction (Section 3.4).

### 3.4 Evolution of the Chemical Composition of Catalysts with ODH Reaction Conditions and Identification of Site A (XPEEM Analysis)

**Figure 10:**
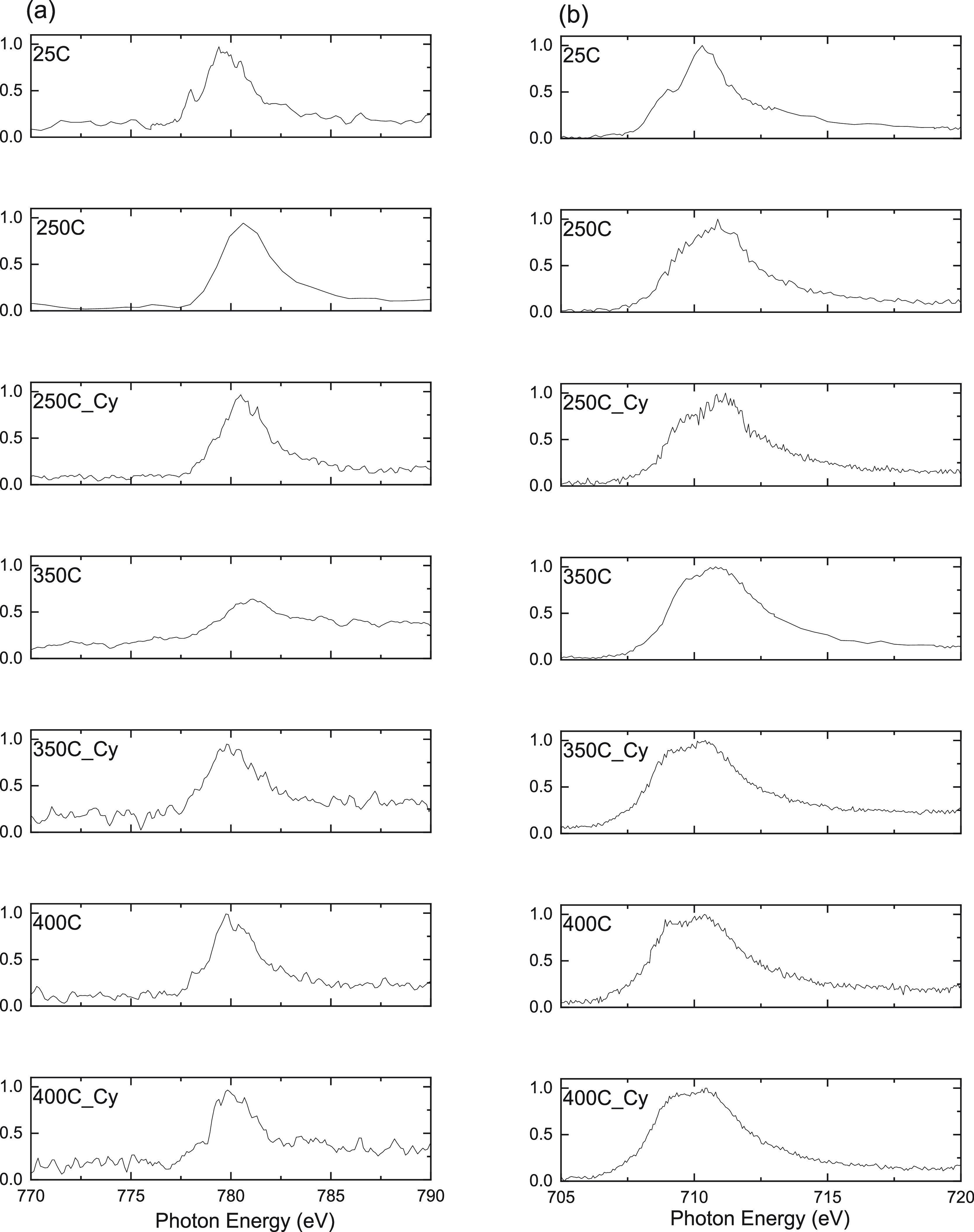
XA spectra of (a) Co and (b) Fe in 7 nm CoFe NPs on RGO (RGO-CoFe1) at each reaction condition. 25, 250, 300, 350, and 400 indicate the reaction temperature in °C within the XPEEM chamber at pressure 1 × 10^–10^ mbar and 250C_Cy, 350C_Cy and 400C_Cy indicates XA spectra recorded after the dosing of cyclohexane and O_2_ in 1:1 ratio at 250, 300, and 400 °C respectively.

The effect of ODH reaction conditions on the chemical state of RGO-CoFe nanohybrids (small, RGO-CoFe1 and large, RGO-CoFe4) is analyzed by XPEEM. Figure 10 shows the evolution of XA spectra acquired from the particle aggregates of RGO-CoFe1 (7 nm 75 wt %) upon exposure to reaction temperatures and cyclohexane and O_2_ in 1:1 ratio at respective temperatures and compared with the pristine state of the NPs at room temperature (25 °C). The XA spectra of Co at 25 °C display a typical structure of Co^2+^/O_h_ compounds where the *L*_3_ edge is characterized by three peaks at 778, 779.3, and 780.3 eV with a shoulder at the high-energy side of the *L*_3_ peak at 783 eV. The XA spectra at the *L*_3_ edge of Fe at 25 °C consists of the main peak at 710.4 eV and a shoulder at 709 eV. Typically, in CoFe_2_O_4_, the Co^2+^/O_h_ cation has strong transition peaks that correspond to the three main *L*_3_ peaks, as observed in Figure 10, whereas the Co^2+^/T_d_ cation has only one strong transition peak that corresponds to the main *L*_3_ peak and a shoulder at the high energy side of the *L*_3_ peak.^62,64^ The observed broad nature of the *L*_3_ peak in Figure 10 is due to the contribution from both O_h_ and T_d_ Co^2+^ cations that occur at slightly different photon energies. In the case of Fe in CoFe_2_O_4_, Fe^3+^/O_h_ cations have strong transitions that correspond to the shoulder at low energy *L*_3_ and the main *L*_3_ peaks. The Fe^3+^/T_d_ peak occurs slightly at the lower energy of the *L*_3_ peak, and it does not have transition corresponding to the shoulder at low-energy *L*_3_ peak like the Fe^3+^/O_h_ cation. The Fe^2+^/O_h_ cation has a strong transition peak corresponding to the low-energy *L*_3_ peak and a weak transition peak at the low-energy *L*_3_ shoulder.^62,64^ Thus, the XA spectral evolution of Co and Fe displayed in Figure 10 is composed of various contributions from these spectral components (Co^2+^, Fe^3+^, and Fe^2+^ cations at O_h_ and T_d_ locations) as a function of reaction conditions. Reference XA spectra of Co^2+^, Fe^3+^, and Fe^2+^ cations at O_h_ and T_d_ locations, required for fitting the experimental spectra using the linear combination method, were not experimentally measured in this work or elsewhere. Therefore, the MCR ALS method was employed to identify (using C_0_ or S^t^) and quantify the fractions of each components (Co and Fe in O_h_ and T_d_ locations) in the measured XA spectra (concentration profiles). We used calculated XA spectra by Moyer et al. using the Ligand Field Model (LFM)^64^ to identify the components (XA spectra of Co^2+^, Fe^3+^, and Fe^2+^ cations at O_h_ and T_d_ locations) produced by MCR ALS (Sections 2.6 and S5).

**Figure 11:**
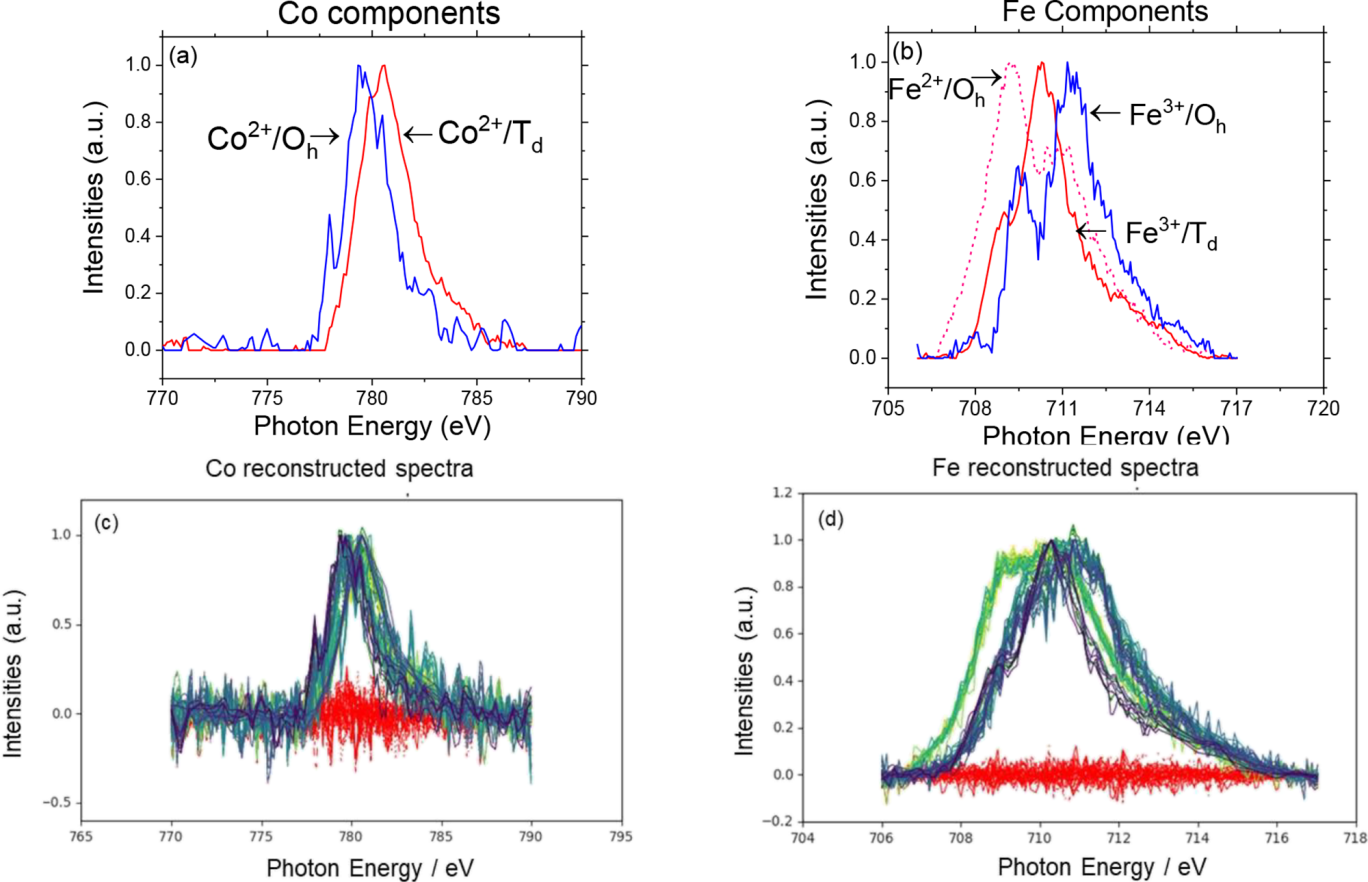
XA spectra of (a) Co components and (b) Fe components acquired from MCR ALS analysis. Figure 11 (c), (d) shows the comparison between the reconstructed and experimentally measured XA spectra at all the reaction conditions for Co and Fe, respectively, for the RGO-CoFe-1 (7 nm CoFe_2_O_4_ 75 wt %) sample. Red lines: residuals E, dotted lines: reconstructed (*X̂*), solid lines: experimental spectra (*X*).

Using MCR ALS, in the case of Co, we show that all the experimental spectra in the RGO-CoFe1 sample at all the ODH reaction conditions can be adequately described by the presence of two distinct components (Figure 11a): First, with the *L*_3_ peak position at 779.3 eV and second at 780.3 eV in both 7 and 12 nm particles. The first peak at 779.3 eV has two sharp spectral features at the lower (778 eV) and higher (780.5 eV) sides of the *L*_3_ peak which indicates the presence of Co^2+^ cations at O_h_ locations^64^ while the other peak at 780.5 eV is assigned to Co^2+^ cations located at T_d_ locations.^64^ Similarly, in the case of Fe, all the experimental spectra in 7 nm NPs can be adequately described by three components, as shown in Figure 11b, with the peaks at 709, 710.3, and 711.5 eV which were assigned to Fe^2+^ in octahedral sites, Fe^3+^ in octahedral sites, and Fe^3+^ in tetrahedral sites, respectively. These peak positions are comparable with the theoretically reported components in CoFe_2_O_4_ by Moyer et al.^64^ and Zhou et al.^65^ (Figure S12).

**Figure 12:**
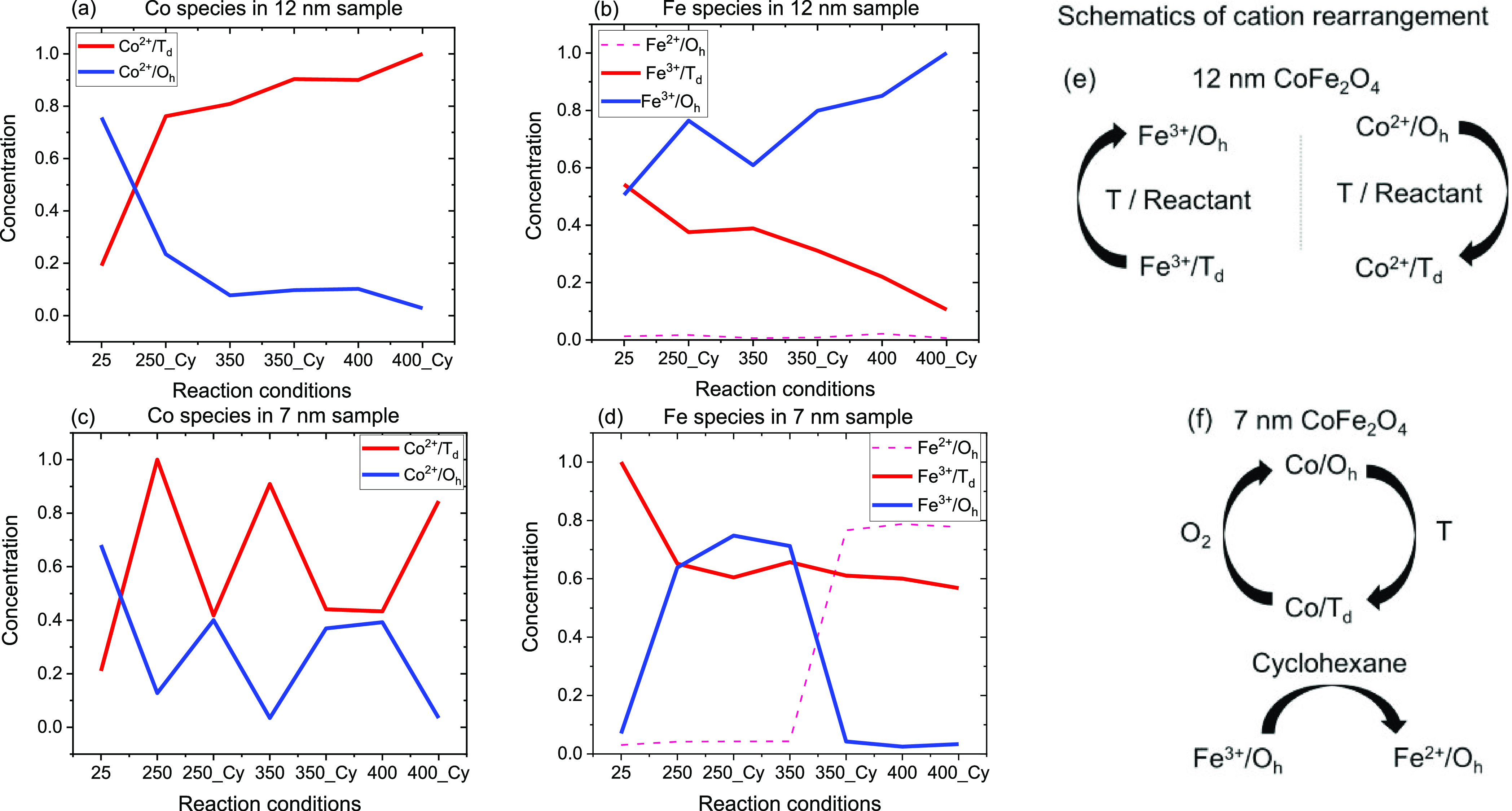
MCR ALS concentration profiles of the spectral components as a function of reaction conditions. 12(a) and (b) displays concentration profiles of Co and Fe cations in 12 nm samples, respectively, 12(c) and (d) displays concentration profiles of Co and Fe cations in 7 nm samples. 25, 250, 300, 350, and 400 indicate the reaction temperature in °C within the XPEEM chamber at pressure 1 × 10^–10^ mbar and 250_Cy, 350_Cy, and 400_Cy indicate the presence of cyclohexane and O_2_ in 1:1 ratio with total pressure around 2 × 10^–5^ mbar at 250, 300, and 400 °C respectively. The concentration profiles are presented in the chronological order as experiments performed and represent an average behavior of six particle aggregates at each condition. See Figures S13 and S14 for the evolution of respective spectra of pure species. Figure 12(e) and (f) displays schematics of cation arrangement in 12 and 7 nm samples, respectively.

These components for Co and Fe and the corresponding concentration profiles (Figure 12) were obtained after reconstructing the XA spectra while minimizing the error between experimentally measured and re-constructed spectra using MCR ALS, as shown in Figure 11c,d (SI Section S5). These concentration profiles represent the relative fractions of the corresponding components within the bulk of the particle that evolve as a function of ODH reaction conditions (see the spectral evolution of the respective species at ODH reaction conditions in Figures S13 and S14). A similar treatment was performed on large NPs (RGO-CoFe4, SI Section S6, Figures S12 and S13). Figure 12 then reveals important characteristics of a dynamic interplay of cations within the bulk of both small and large NPs during the reaction and how it depends on the size of the CoFe_2_O_4_ nanoparticle. The concentration profiles for Co and Fe cations in 12 nm particles are displayed in Figure 12a,b and those in 7 nm particles are displayed in Figure 12c,d, respectively. The corresponding dynamic nature of these cations is schematically displayed in Figure 12e,f for 12 and 7 nm particles, respectively.

#### 3.4.1 At 25 °C (12 and 7 nm Particles)

Initially, at 25 °C, both 12 and 7 nm particles showed similar concentrations of Co^2+^ species with a higher relative abundance of Co^2+^/O_h_ species (Figure 12a,c, respectively). The relative concentrations of Fe^3+^/O_h_ and Fe^3+^/T_d_ at 25 °C, however, were different on both sizes. The large particle (Figure 12b) exhibited similar relative concentrations of Fe^3+^/O_h_ and Fe^3+^/T_d_ species while the smaller particle (Figure 12d) showed higher relative population of Fe^3+^/T_d_ species 25 °C.

#### 3.4.2 At Higher Temperatures (12 nm)

In the case of 12 nm sample, we further observe that the population of Co^2+^/O_h_ species decreases while the population of Co^2+^/T_d_ species increases as a function of temperature and the presence of reactants (Figure 12a). This might suggest the migration of Co^2+^ cations from O_h_ to T_d_ sites as a function of reaction temperature. Such migration would then create O_h_ site-vacancies which are then appeared to be filled by Fe^3+^ cations which migrate from their T_d_ sites to O_h_ (Figure 12b). Such preference of Fe^3+^ cations for octahedral coordinations over Co^2+^ cations^66^ and temperature effect on cationic coordination exchange in CoFe_2_O_4_ was previously documented^67^ and consistent with this work. Not many differences in the population of these species were observed when the reactants were introduced at the same temperature, suggesting that the temperature is solely responsible for the coordination exchange and second, the catalyst particles exhibit Co^2+^/T_d_ and Fe^3+^/O_h_ as the most abundant species on the surface as well as in the bulk of 12 nm particles. The relative population of Fe^2+^/O_h_ species remained low at all reaction conditions (Figure 12b). This interexchange of coordinations between Co and Fe cations is shown schematically in Figure 12e, suggesting that these species (Co^2+^/T_d_ and Fe^3+^/O_h_) could be potential active sites for O_2_ dissociation in ODH of cyclohexane in the case of 12 nm particle.

#### 3.4.3 At Higher Temperatures (7 nm)

In the case of 7 nm CoFe_2_O_4_ particles, the situation is more complex. Unlike 12 nm particles, the small particles showed significant dependence on the presence of reactants and the populations of Co O_h_ and T_d_ species responded accordingly. Examining the concentration profiles in Figure 12c,d, we propose that as the temperature increases (e.g., from 25 to 250 °C, without reactants) Co^2+^ ions migrate from O_h_ position to T_d_ position creating O_h_ site-vacancies, consistent with the behavior of 12 nm particles. These O_h_ site-vacancies are, however, not occupied by Fe^3+^ cations and therefore remain vacant. Upon introducing the reactants (cyclohexane and O_2_ in 1:1 ratio), some of these Co^2+^/T_d_ species (probably on the particle surface) again migrate back to O_h_ locations, supposedly due to additional O-species that can form on Co upon O_2_ dissociation, as previously suggested.^30,31^

This cycle of Co^2+^ cation migration from O_h_ to T_d_ locations appeared to be continued until 350 °C (350_Cy, Figure 12c) without the participation of Fe^3+^ cations. It is worth pointing out that, at 350 °C, in the presence of reactants (350_Cy), the resulting final concentration of Co^2+^/T_d_ and Co^2+^/O_h_ species (at 350_Cy) is consistent with the XPS analysis (Figure 9b, 13 wt % used sample) as revealed by complementary XPS (surface sensitive) and XA (bulk sensitive) analysis and indicate that this atomic interplay probably reflects the activity at the surface of the particle.

We argue the reasons that the Fe^3+^ cations did not participate in this interplay as follows: Initially, up to 250 °C, the Fe^3+^/T_d_ cations do migrate to O_h_ locations as observed by decrease and increase of corresponding concentrations, in Figure 12d. At higher temperatures, however, in contrast to the large particles, the decrease in the concentration of Fe^3+^/T_d_ is not significant. This allowed us to propose that the initial observed decrease in Fe^3+^/T_d_ cations concentration is mostly due to the migration of the Fe^3+^/T_d_ cations to O_h_ locations in the bulk of the particle. The latter marginal decrease in Fe^3+^/T_d_ concentration might be because the surface Fe^3+^/T_d_ cations could be strongly bound to surrounding RGO support, as indicated by XPS analysis. Noting the stability of the RGO support from the TGA data approves the fact that RGO could remain intact and wrap the smaller NPs efficiently through Fe^3+^/T_d_ as a contact point. This wrapping of RGO could mask Fe^3+^/T_d_ cations and expose Fe^3+^/O_h_ cations which then react with cyclohexane and derived moieties and may reduce to Fe^2+^/O_h_ cations due to H-abstraction from cyclohexane. Note that the XPS analysis did not detect the presence of Fe^2+^/O_h_ cations on the NPs surface after the ODH reaction which indicates that such reduction of Fe^3+^/O_h_ to Fe^2+^/O_h_ only took place under the high vacuum reductive environment in the XPEEM chamber even in the presence of O_2_, and at actual reaction conditions (350 or 400 °C, *P*_cyclohexane_ = *P*_O2_ = 0.2 kPa), Fe^3+^/O_h_ did not reduce to Fe^2+^/O_h_.

On the other hand, the high benzene selectivities observed on this sample (RGO-CoFe1 and 4, Figure 4b) and high abundance of Fe^3+^/T_d_ sites (XPS, Figure 9a) and higher predicted affinity of these sites to bind RGO incline us to propose that the Fe^3+^/T_d_ cations could also bind cyclohexane and cyclohexane-derived species formed during the reaction given their similar chemical structure (sp2 hybridized carbon) with RGO. In this case, such strong Fe-cyclohexane interaction^11^ could suggest that Fe^3+^ cations at T_d_ locations are probably responsible for the full dehydrogenation of cyclohexane to benzene. It is worth noting that in the 12 nm sample, the Fe^3+^/T_d_ population remained low probably because most of the Fe^3+^/T_d_ cations migrated to O_h_ locations. This explains the observed higher cyclohexene selectivity (31%) on the large NPs at higher wt % loading (12 nm, 76 wt %, Figure 4b) compared to 7 nm 75 wt % sample. Thus, observing these concentration profiles collectively, we anticipate that the Fe^3+^ cations at T_d_ locations might be mainly responsible for full dehydrogenation to benzene formation. These Fe^3+^/T_d_ sites can either bind to RGO if the particle size is smaller or they can migrate to O_h_ locations if the particle size is larger; thus, these catalysts represent the unique way to re-orient the populations of these sites on the surface of the nanoparticle.

In brief, we observed a dynamic interplay between Co and Fe cations exchanging their respective locations during the reaction. For 12 nm particle, unlike 7 nm particle, the Fe^3+^/T_d_ species migrate to vacant O_h_ locations formed due to the concomitant migration of Co^2+^/O_h_ species to T_d_ locations. Thus, in the 12 nm particles, most of the Co^2+^ cations are at T_d_ locations and Fe^3+^ cations are at O_h_ locations. The 7 nm particles, however, can be wrapped by the RGO support to a higher extent compared to 12 nm particles (consistent with XPS and kinetic analysis above). Examining the concentration profiles of Fe species in the 7 nm particles, we anticipate that the surface Fe^3+^/T_d_ species may specifically bind to RGO and are blocked; thus, they are not able to migrate and occupy the vacant sites created by the migration of Co^2+^/O_h_ species to T_d_ locations.

Examining the concentration profiles of Fe^3+^/T_d_ species and how it is affected by the reaction temperature and reactants and their relative high abundance (Figure 12), we conclude that Fe^3+^/T_d_ could represent site A that the kinetic studies suggest as discussed above. Thus, the CoFe_2_O_4_ nanoparticle surface could exhibit bare Fe^3+^/T_d_ sites and O* species during the reaction and their relative population decide which pathway the ODH reaction could follow which in turn can be controlled by varying wt % contents of CoFe_2_O_4_ NPs. We show that in the 12 nm particles, the Fe^3+^/T_d_ sites quickly exchange their coordination with Co^2+^/O_h_ species as discussed above (Figure 12). The 7 nm particles, on the other hand, appear to be specifically bound to the RGO support via Fe^3+^/T_d_ if it is in direct contact with it (13 wt %) (Figures 9 and 12); otherwise, it exhibits a higher fraction of Fe^3+^/T_d_ species (75 wt % sample) which is then used to drive the reaction toward complete dehydrogenation to form benzene.

Next, the kinetic analysis shows how such cationic distribution can be used to manipulate the abundance of site A and site B in order to enhance the cyclohexene selectivity by promoting its desorption before it undergoes subsequent dehydrogenation reactions by precisely eliminating the (Fe^3+^/Td) sites on the surface of the nanoparticle by varying the loading of CoFe_2_O_4_ particles on the RGO support.

### 3.5 Mechanistic Insights into Cyclohexane Oxidative Dehydrogenation in a Single Surface Visit: Role of Site A (Fe^3+^/T_d_) and Site B (O*)

**Figure 13:**
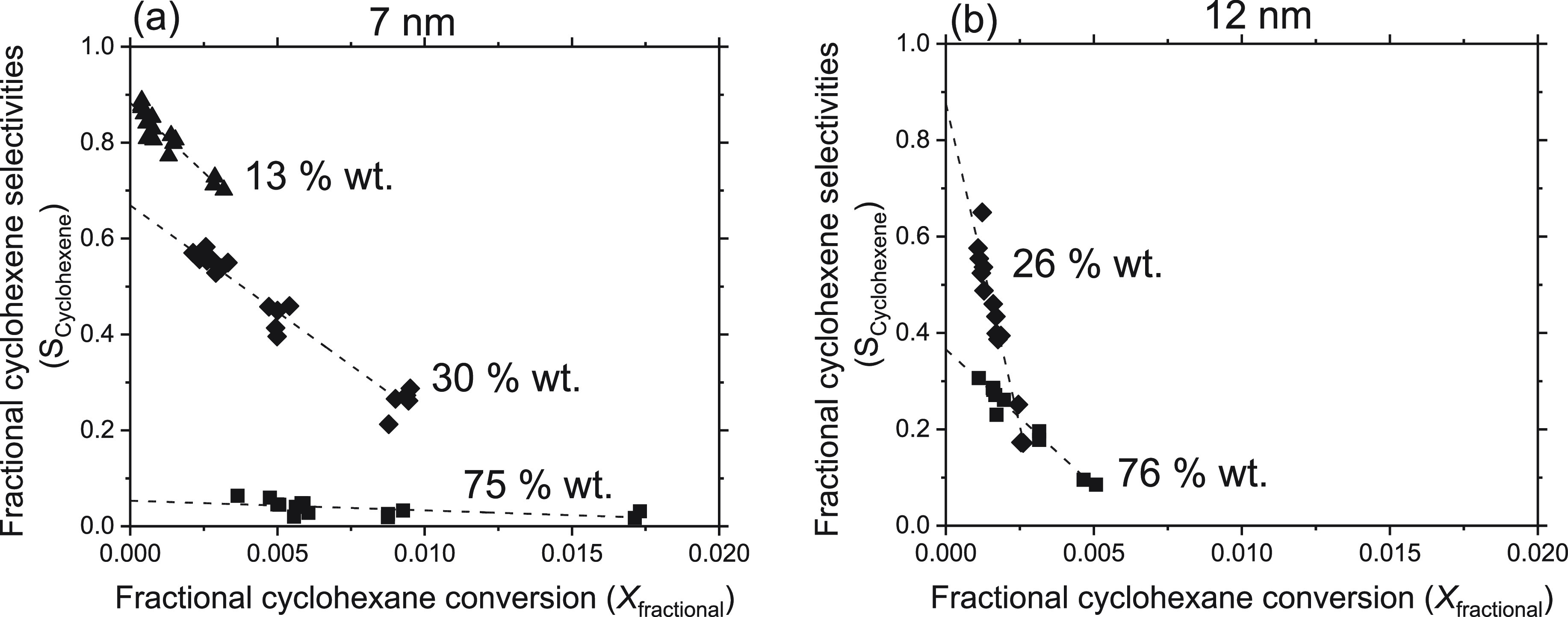
Fractional cyclohexene selectivities as a function of fractional cyclohexane conversion measured on 7 (a) and 12 nm (b) CoFe_2_O_4_ particles with various loadings (in wt %) on RGO measured at 350 °C and *P*_cyclohexane_ = *P*_O2_ = 0.2 kPa. The dashed lines are the best fit to eq 6.

**Scheme 1:**
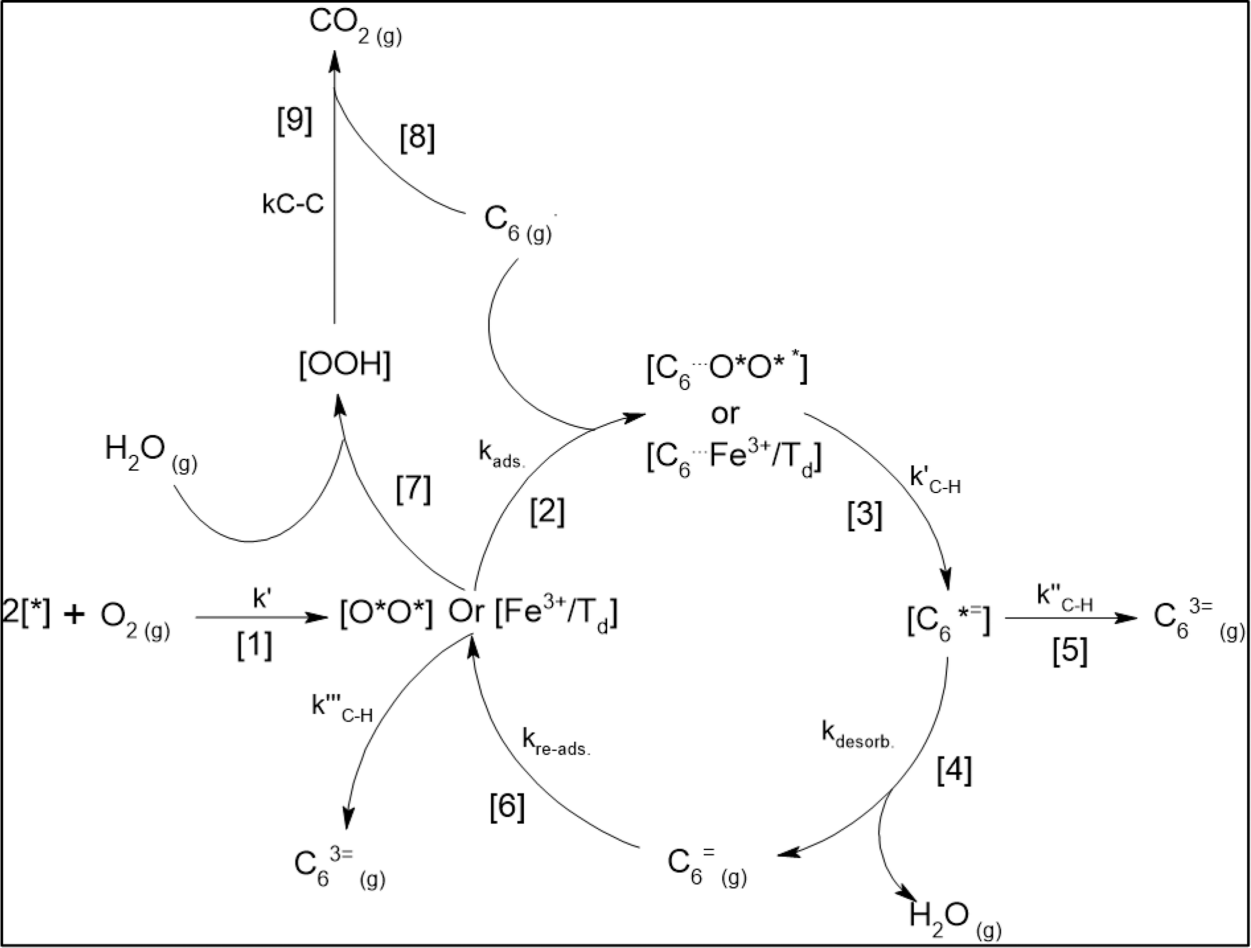
Elementary Steps in Cyclohexane ODH Reactions on CoFe_2_O_4_ Nanohybrids

These observations in Sections 3.3 and 3.4 taken together with the selectivity-conversion dependence of cyclohexene, benzene, and CO_2_ (Figure 13) indicate that the cyclohexane oxidative dehydrogenation proceed via elementary steps shown in Scheme 1, with the first C–H bond dissociation of cyclohexane as the sole kinetically relevant step on O*-saturated surfaces (step 3) consistent with the previously shown DFT analysis.^11^ The elementary steps shown in Scheme 1 involve the formation of O* species on the bare metal centers (*, Co^2+^/T_d_ and/or Fe^3+^/O_h_) and the *k*_app-O2_ indicate the apparent O_2_ dissociation constant which contain O_2_ molecular adsorption on (*) and followed by dissociation forming O*O* species (Step 1) consistent with recent DFT studies.^30,31^ Adsorption of cyclohexane on O*O* (or on Fe^3+^/T_d_) (step 2, *K*_ads_) followed by C–H dissociation (step 3, *k*′_C–H_) may form cyclohexyl radical like species (C_6_*^=^) which may desorb as cyclohexene (C_6_^=^_(g)_, *k*_desorb_, Step 4) or undergo further subsequent dehydrogenation (*k*″_C–H_, step 5) to form benzene (C_6_^3=^_(g)_) in a single catalytic visit. The abundance of O* or Fe^3+^/T_d_ on the NP surface, would decide the fate of cyclohexane-derived species to desorb as cyclohexene or undergo subsequent dehydrogenation to form benzene. In the latter case, cyclohexane adsorption would occur on Fe^3+^/T_d_ which would undergo concomitant subsequent dehydrogenation forming benzene. Note that cyclohexadiene was not detected in the experimental conditions used in this work; therefore, *k*″_C–H_ combines the rate constants for all the remaining C–H bond dissociation until benzene formation occurs. The desorbed cyclohexene can re-adsorb on O*O* sites (or Fe^3+^/T_d_ sites) and undergo further dehydrogenation in a second catalytic visit forming benzene (step 6, *k*_re-adsorb_).

It is worth noting that the selectivity of CO_2_ in this work did not depend on the changes in the reactor resident times for all the catalysts studied here, neither its rates of formation depended on the wt % loading (Figure S8). Such behavior strongly indicates that the active sites and the intermediates involve in the formation of CO_2_ are different from those involved in the dehydrogenation reactions which we surmise to be OOH species^58^ without further evidence. The formation of OOH species could occur by reaction between O* species and water formed during the ODH reaction (step 7, *k*_OOH_) followed by their consumption by cyclohexane (step 8) to form CO_2_ (step 9, *k*_C–C_) via C–C bond cleavage through O-insertion. We show that in this way, the coupled differential equations that account for the CO_2_ formation, as shown in Scheme 1, are essentially residence-time-independent and thus may indicate autocatalysis in the formation of CO_2_, as ODH reaction by-product (H_2_O) is responsible for the formation of the active sites (OOH) (Supporting information Figure S8, Section S6). In addition, it is worth mentioning that the recent theoretical work by Avci et al.^68^ on CoFe_2_O_4_ for O_2_ evolution reactions predicts the formation and stability of OOH species specifically on Co atoms, which support the findings of this work.

We investigate in detail what happens to cyclohexane molecules in a single visit on the catalytic surface by examining the cyclohexene selectivity dependence on cyclohexane conversion on different wt % contents of CoFe_2_O_4_ particles on RGO. The presence of two kinds of active sites with relatively different reactivities toward C–H bond cleavage could result in either specific desorption of cyclohexene (*k*_desorb_) or subsequent C–H bond cleavage (*k*″_C–H_) to form benzene in a single surface visit and thus confirming their presence and relative abundance.

Figure 13 shows the selectivity of cyclohexene (eq 6) at different levels of cyclohexane conversion (*X*). The cyclohexane conversion is varied by changing the residence times at similar cyclohexane and O_2_ partial pressures (*P*_cyclohexane_ = *P*_O2_ = 0.2 kPa) for different wt % loadings. The cyclohexene selectivities extrapolated to zero conversion reflect the extent to which cyclohexyl species formed in a primary C–H bond cleavage event desorb as cyclohexene before undergoing subsequent C–H bond cleavage to full dehydrogenation. Thus, such asymptotic selectivities at zero conversion immediately confirm which site cyclohexane is adsorbed on, which in turn indicates their relative abundance on the surface of the particle in a given wt % sample.

These asymptotic selectivities extrapolated to zero conversion are less than unity and decrease with increasing wt % of CoFe_2_O_4_ NPs on RGO, indicative of rates of subsequent C–H bond cleavage (*k*″_C–H_, step 5) in [C_6_*^=^] species increase as wt % content of NPs increase. It also suggests the increased abundance of sites A (Fe^3+^/T_d_) with increasing wt % content. The lowest wt % (13 wt % for 7 nm and 26 wt % for 12 nm) samples leads to asymptotic cyclohexene selectivities near unity at zero conversion, consistent with the increase in preferential desorption rates (*k*_desorb_, step 4) over subsequent C–H bond cleavage events (*k*″_C – H_, Step 5). The cyclohexene selectivities decrease with conversion (Figure 13) in all cases because cyclohexene can re-adsorb and undergo subsequent C–H bond activation events that form benzene; step 6. Scheme 1. The slopes in Figure 13 account for the readsorption of cyclohexene formed in the first event and its subsequent C–H bond cleavage to form benzene.
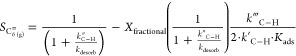
6

**Figure 14:**
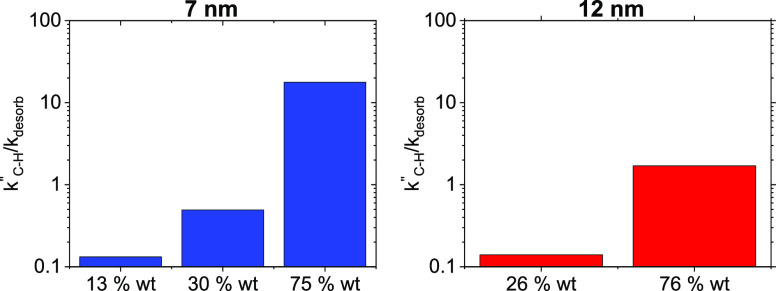
Ratio of  extracted from Figure 13 after fitting to eq 6 and plotted as a function of wt % loading of (a) 7 nm and (b) 12 nm CoFe_2_O_4_ particles deposited on RGO.

The rate constants for steps 3–5 (Scheme 1) account for the slope and intercept of the cyclohexene selectivities, as shown in Figure 13, in a manner that is consistent with the plug-flow reactor description of the rates of primary and secondary events along the bed. The resulting coupled differential equations account for the rates of cyclohexene and benzene formation and include the rate equations derived from the elementary steps in Scheme 1 (details in the Supporting Information Section S6). The dashed lines in Figure 13 are the best fit to eq 6 and the ratio of  was extracted and plotted in Figure 14 as a function of different wt % loadings in 7 and 12 nm particles. The estimated small values of the ratios of  indicate that cyclohexene is the exclusive product formed on the samples with lower loadings (13 wt % for 7 nm and 26 wt % 12 nm) in a single catalytic event which desorbs before undergoing subsequent dehydrogenation. The increase in the value of the ratio  with increasing wt % content of CoFe NPs renders that the rates for subsequent dehydrogenation reactions (*k*″_C – H_) are higher on high wt % loading samples. This observation is consistent with the prediction of two types of sites on CoFe particles whose relative abundance is varied by varying the wt % of NPs on RGO.

On the higher wt % samples, the particles are crowded and in direct contact with other particles with high abundance of Fe^3+^/T_d_ species (site A) exposed to cyclohexane reactant. Such crowded surface makes RGO support unable to access and mask Fe^3+^/T_d_ sites as it would in case of less-crowded surfaces. Thus, at higher wt % samples, we specifically expose these Fe^3+^/T_d_ sites in higher abundance which has higher affinity towards cyclohexane and derived intermediates. Therefore, cyclohexane undergoes full dehydrogenation on these sites as observed in Figures 13 and 14. On the contrary, these aforementioned sites can efficiently bind with RGO support in samples with lower wt % because these facets are in close contact with RGO (for 7 nm) or migrate to O_h_ location (for 12 nm) thereby decreasing their relative abundance. Therefore, cyclohexane undergoes only partial dehydrogenation forming cyclohexene on O* species (site B) which predominantly cover the surface.

The slopes in Figure 13 are accounted for by the ratio  which compares the rate constant of C–H bond abstraction in cyclohexene upon its re-adsorption (*k*‴_C – H_) to the product of the rate constant for first C–H bond abstraction in cyclohexane and its adsorption equilibrium constant (*k*′_C – H_ · *K*_ads_). The larger magnitudes of the slopes observed for smaller wt % samples (13 wt %, 30 wt % for 7 nm and 26 wt % for 12 nm) indicates that the C–H bond abstraction in cyclohexene is easier and faster than that in cyclohexane on O* sites. The desorbed cyclohexene molecule can re-adsorb on O* species which are abundant on the lower wt % samples and can undergo further dehydrogenation to form benzene. The much smaller magnitudes of the slopes observed on higher wt % loading samples (75 wt % on 7 nm and 76 wt % on 12 nm) further indicates rather stronger adsorption (*K*_ads_) and faster C–H bond abstraction (*k*′_C – H_) of cyclohexane compared to O* species. Thus, these cyclohexane-derived moieties remain strongly adsorbed preferentially on Fe^3+^/T_d_ sites and undergo full dehydrogenation, consistent with the pressure dependence studies shown in Figures 6 and 7.

Our complementary evidence obtained through kinetic and spectroscopic investigation consistently suggests the presence of two types of active sites exhibited by CoFe_2_O_4_ particles on the RGO support. Thus, by gradually varying the wt % loading of CoFe_2_O_4_ particles on the RGO support, we successfully manage to expose one of these two sites in abundance systematically, thus achieving full control over reaction selectivity (cyclohexene and benzene) without affecting overall rates. Varying the wt % loading allowed us to gradually eliminate the Fe^3+^/T_d_ sites either by masking them with RGO or migrating them Oh locations. Such consequences of support effects on cationic coordination interplay, resulting in exposing different active sites as desired extends beyond oxidative dehydrogenation reactions, suggesting that such an approach could be versatilely employed in reactions which suffer selectivity control due to thermodynamic limitations.

## 4 Conclusions

Here, we conduct a thorough investigation of cyclohexane oxidative dehydrogenation (ODH) catalyzed by cobalt ferrite (CoFe_2_O_4_) nanoparticles supported on RGO. Using spectroscopic (XPEEM and XAS in ex-situ) and kinetic analysis, we show that CoFe_2_O_4_/RGO nanohybrids possess two types of active sites that have different relative affinities toward cyclohexane-derived intermediates. We postulate that Fe^3+^/T_d_ species possess high affinity toward cyclohexane and related intermediates and are responsible for complete cyclohexane dehydrogenation to benzene in a single catalytic sojourn. Additionally, we postulate that oxygen-derived species (O*) are responsible for partial cyclohexane dehydrogenation to form cyclohexene in a single sojourn. We reveal that these NPs when deposited on the RGO support undergo dynamic structural changes as a function of reaction conditions but only when these particles are not crowded on the RGO support, and there is sufficient contact between RGO and NPs (low wt % loading), thus suggesting a unique role of the support. In the case of large NPs (12 nm), the Fe^3+^/T_d_ cations migrate to octahedral locations followed by concomitant migration of Co^2+^/O_h_ cations to tetrahedral sites. In the case of small NPs (7 nm), we hypothesize that RGO support could specifically bind to Fe^3+^/T_d_ sites, thus masking their exposure to the reactants, also preventing them migrate to Oh locations. Thus, in small NPs, Co cations interexchange the positions from O_h_ to T_d_ and back to O_h_ as a function of reaction temperature and the presence of reactants, respectively. Additionally, we also reveal that these NPs are strongly bound to the RGO support, thus preventing them from possible agglomeration or collapsing of the mixed-oxide spinel structure into two different phases (Fe_3_O_4_ and Co_2_O_3_) as a function of reaction conditions, an effect observed on unsupported NPs. We use these phenomena and vary the wt % content of CoFe NPs on RGO gradually which allowed us to gradually expose either Fe^3+^/T_d_ or O* species, thus finally managing to control the reaction selectivity uniquely to benzene or cyclohexene. The present catalysts, composed of inexpensive and abundant crustal metals such as Fe and Co, are demonstrated to be highly efficient, economic, and with high life-time (∼100 h) catalysts, also selective to the sustainable production of fuels and chemicals, thus offering a potential solution to an important and challenging target conversion reaction.

## Data Availability

The metadata and data that supports the findings of this study are available from the corresponding authors upon reasonable request.

## Supporting Information Available

TEM images of the unsupported CoFe particles; TGA analysis; additional kinetic data; C 1s and O 1s XPS spectra of fresh and spent catalysts (7 nm, 75, and 12 wt %); XPEEM image; XAS data acquisition; chemometric analysis; derivations of the rate expressions; and selectivity–conversion relation (PDF)
