# Supplementary material for: Cyclohexane Oxidative Dehydrogenation on Graphene-Oxide-Supported Cobalt Ferrite Nanohybrids: Effect of Dynamic Nature of Active Sites on Reaction Selectivity
Source: ACS Catal. 2023 Oct 5;13(20):13484–505. doi: 10.1021/acscatal.3c02592 (PMC10594591; doi:10.1021/acscatal.3c02592)
Supplement: Supplementary file 1 — cs3c02592_si_001.pdf [file cs3c02592_si_001.pdf]

Supporting Information Section

Cyclohexane oxidative dehydrogenation on graphene oxide supported cobalt ferrite nanohybrids: Effect of dynamic nature of active sites on reaction selectivity

Shashikant A. Kadam*[1 €], Stefania Sandoval [2 €], Zdeněk Bastl [2], Karolína Simkovičová [1], Libor Kvítek [3], Juraj Jašík [1], Joanna Elżbieta Olszówka [1], Stanislav Valtera [1], Mykhailo Vaidulych [1], Jaroslava Morávková [4], Petr Sazama [4], David Kubička [5], Arnaud Travert [6], Jeroen A. van Bokhoven [7], Alessandro Fortunelli [8], Armin Kleibert [9], Martin Kalbáč [2], and Štefan Vajda* [1]

[1] Department of Nanocatalysis, [2] Department of Low Dimensional Systems and [4] Department of Structure and Dynamics in Catalysis, J. Heyrovsky Institute of Physical Chemistry of the Czech Academy of Sciences v.v.i, Dolejškova 3, 18223, Prague, Czech Republic

[3] Department of Physical Chemistry, Faculty of Science, Palacký University Olomouc, 17. Listopadu 12, 77900 Olomouc, Czech Republic

[5] University of Chemistry and Technology Prague, Department of Petroleum Technology and Alternative Fuels, Technická 5, 166 28 Prague, Czech Republic

[6] Normandie Univ., ENSICAEN, UNICAEN, CNRS, Laboratoire Catalyse et Spectrochimie, 14000 Caen, France

[7] ETH Zürich, Vladimir-Prelog Weg 1, Zürich 8093, Switzerland

[8] CNR-ICCOM, Consiglio Nazionale delle Ricerche, via G. Moruzzi 1, Pisa, Italy

[9] Swiss Light Source, Paul Scherrer Institut, 5232 Villigen PSI, Switzerland

* Email: shashikant.kadam@jh-inst.cas.cz and stefan.vajda@jh-inst.cas.cz

Section S1: Particle size distribution of small and large nanoparticles and XRD pattern

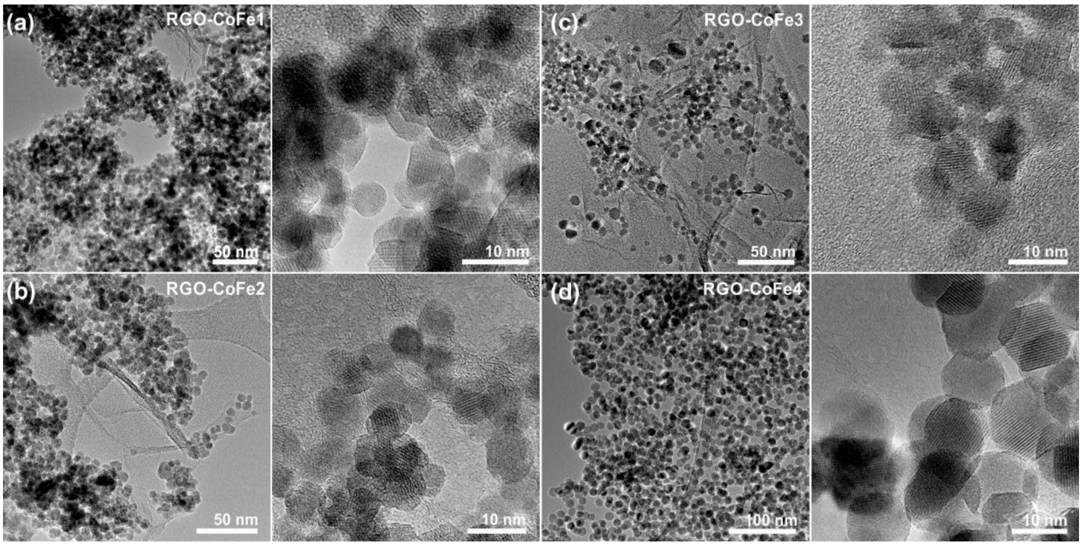

Fig. S1. TEM micrographs of RGO-$CoFe_2O_4$ based catalysts prepared by anchoring (a-c) small and (d) large $COFe_2O_4$ NPs onto graphene oxide supports.

After measuring ca. 200 nanoparticles, Gaussian-like size distributions were observed (mean diameter = 6.7 nm ± 1.3 nm and 12.3 nm ± 1.7 nm, respectively) for both S-CoFe and L-CoFe NPs.

Section S2. TGA Analysis

The TGA of the GO support (black line in Fig. S2) shows two thermal weight loss events at temperatures ca. 200 ºC and 450 ºC respectively. The initial thermal weight loss event (at T ~ 150 – 200°C), which corresponding to the elimination of labile oxygen containing groups (hydroxyls, bridging oxygen moieties, physiosorbed water, etc.) and caused 39.8 % wt decrease in the samples mass at ca. 200 ºC. The final weight loss (59.7 % wt), account for the elimination of more stable aliphatic species such as, aldehydic, ketonic or alcohol groups, present on GO support. At temperature ca 400 ºC these stable moieties are removed followed by the complete oxidation of the sp^2 conjugated C-C bonds of GO at ca. 450 ºC. The TGA of RGO-CoFe 1-3 nanohybrids is significantly different than that of GO support confirming the successful functionalization of these particles on the GO support and their high thermal stability. The initial thermal weight loss event at ca. 200 ºC that was observed on GO support is almost completely absent from the TGA of all the nanohybrids indicating that the oxygen functional groups of GO are now engaged in holding the CoFe particles to the GO support. The only thermal weight loss event for the CoFe nanohybrids at ca. 450 ºC occurs at slightly lower temperatures than GO which is expected, and according to previous reports, this decrease in temperature is proportional to the NPs loading [1, 2] as CoFe favours slight modifications of the GO support. The TGA of RGO-CoFe1-3 confirmed the degree of functionalization of CoFe particles on GO support and the % wt loading of CoFe NPs on GO support was obtained from figure S2 (RGO-CoFe1 (red continuous line), RGO-CoFe2 (blue continuous line) and RGO-CoFe3 (pink continuous line)). Similar observations were recorded for L-CoFe NPs on GO (Fig. S3).

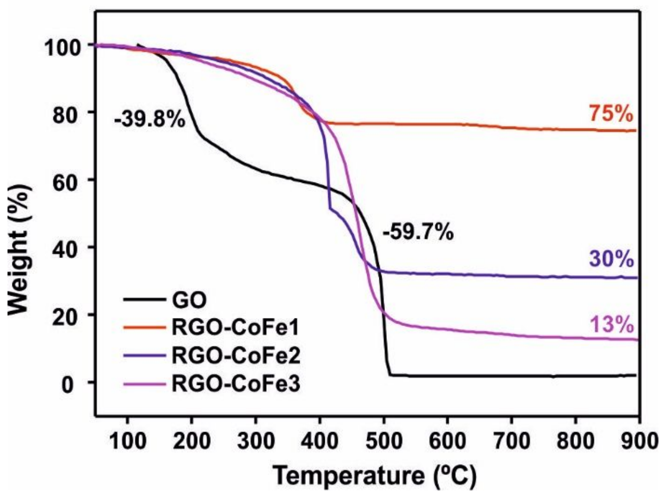

Figure S2. Thermogravimetric analysis of GO and the hybrid nanocomposites prepared by attaching small $CoFe_2O_4$ NPs onto the GO surface.

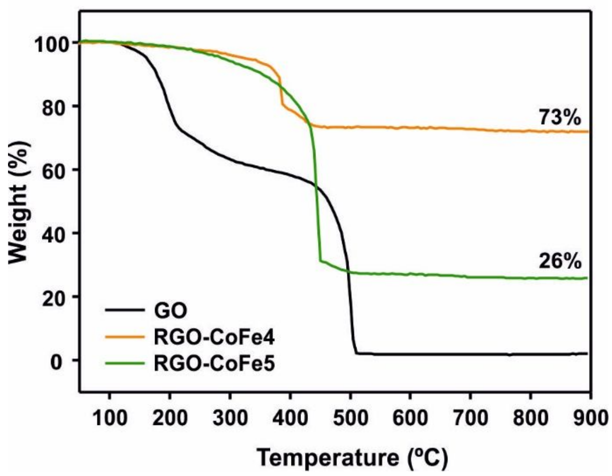

Fig. S3. Thermogravimetric analysis of GO and the hybrid nanocomposites prepared by attaching big $CoFe_2O_4$ NPs onto the GO surface.

Section S3.1: Catalyst pre-treatment

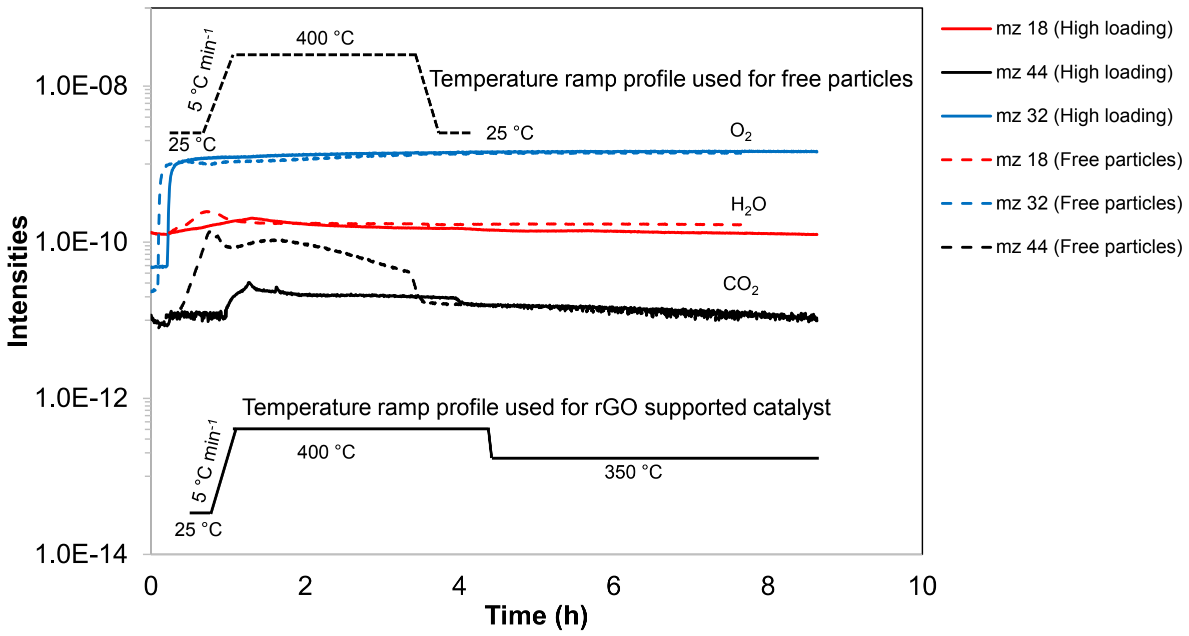

Fig. S4. The m/z profiles for O_2 (m/z 32), H_2O (m/z 18) and CO_2 (m/z 44) during the pre-treatment of 13 nm unsupported (free particles, - - - dashed lines) and 13 nm 75 % wt loading catalyst (solid lines) with the corresponding temperature ramps above for free particles and below for rGO supported particles. Catalysts mass ~ 0.0002-0.0005 g. P_{O2} = 0.2 kPa.

The catalysts pre-treatment under O_2 at 400 °C was monitored by Mass spectrometry. The m/z signals for CO_2 and H_2O increased during the temperature ramp in the temperature range of 100 – 400 °C and then decreased and remained stable, closer to their initial value before the temperature ramp indicating that catalysts surfaces were clean of any organic residues or pre-adsorbed water detectable in mass spectra and high stability of RGO support under the reaction conditions.

The m/z profile for CO_2 clearly shows that the unsupported particles produce a larger amount of CO_2 than the supported ones. This is because the oleic acid moieties used for the synthesis of these nanoparticles still remained on the surface of these particles. However, when these particles are grafted on the rGO support using solvothermal conditions (Table 1, main text), these moieties are essentially removed thus, as evident by lower CO_2 production during the catalyst pre-treatment. The CO_2 intensity remained lower and closer to the initial value even at higher temperature (350 °C) indicating the stability of rGO support under reaction conditions.

Section S3.2. Conversion dependence on reactant residence time

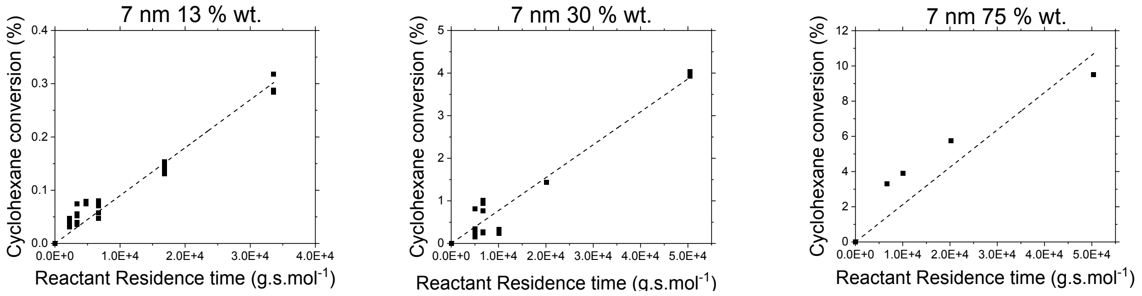

Fig. S5. Cyclohexane conversion as a function of residence time (g_{cat}.s· $mol_{Cyclohexane}^{-1}$) at 350 °C at all % wt loadings of 6 nm particles on rGO. The linear nature of the above curves suggests the absence of mass transfer corruptions.

Section S3.3. Coverage of O* species

These differences in selectivities in Fig. 4b in the main text might indicate the differences in the O* coverages such that the reaction occurs on either O*O* site pairs or at O*-* site pairs where * denote an exposed bare metal center (Co or Fe) as shown previously for Co_3O_4 [3], Ag [4] and Pt [5] clusters. The former site pair can activate C-H bonds in alkane via homolytic hydrogen abstraction steps that form

alkyl species with significant radical character and weak interactions with the surface at the transition states [3-5] followed by consecutive abstraction of second H and releasing alkene, whereas, the latter site pair activates C-H bond heterolytically via oxidative addition reactions that typically involve σ bond metathesis reactions forming metal-carbon σ bonds and involving strong interactions with the surface [3, 4]. The strong metal-carbon interaction in the latter case often led to consecutive and subsequent hydrogen abstraction reactions to produce fully dehydrogenated product over desorption of primary dehydrogenated product. Thus, these two scenarios representing different kinetic regimes with respect to O* coverage and expected to occur at surfaces saturated with O* typically at high O_2 pressures resulting in adjacent O*O* site pairs and at surfaces not saturated with O* at low O_2 pressures exposing bare metal centers resulting in O*-* site pairs, respectively. These two kinetic regimes can also be created by keeping the O_2 pressure constant (0.2 kPa) and varying the number of $CoFe_2O_4$ particles on rGO gradually by varying the % wt loadings. Therefore, the relative surface coverage of O* species could be higher on lower loading sample and vice versa. Thus, based on the observed selectivities in Fig. 4b, we can expect that at lower % wt loading the O*O* as most abundant species producing cyclohexene in high selectivity via homolytic cleavage of C-H bonds, while, at higher % wt loading, O*-* can represent the most abundant active sites producing benzene via heterolytic oxidative addition as expected. This means, by increasing O_2/Cyclohexane ratio, the kinetic regime can be altered from O*-* to O*O* as the most abundant active sites on the samples with high % wt loading of $CoFe_2O_4$ and as a result we may expect increase in cyclohexene selectivities with increasing abundance of O*O* species. Surprisingly, the selectivities toward cyclohexene and benzene were found to be insensitive to O_2/Cyclohexane ratios (in the range of 1 to 25) (See figure S6 below). Such low cyclohexene selectivity can no longer be explained by different kinetic regime at such high O_2 : Cyclohexane ratios leading to high relative O* coverage as expected. In order to understand such insensitivity of cyclohexene selectivity towards oxygen pressure, we consider the stability of O* species formed on the catalyst surface which can have direct impact on selectivities because less strongly the O* are bound to the surface more effective they are in H-abstraction [3, 4]. The highly active O* species (possibly formed on high loading and unsupported particles) can be rapidly consumed by cyclohexane-derived species to form benzene via full dehydrogenation reaction in a single sojourn. In the next session we discuss the stability of O* species and their consumption rates on each sample.

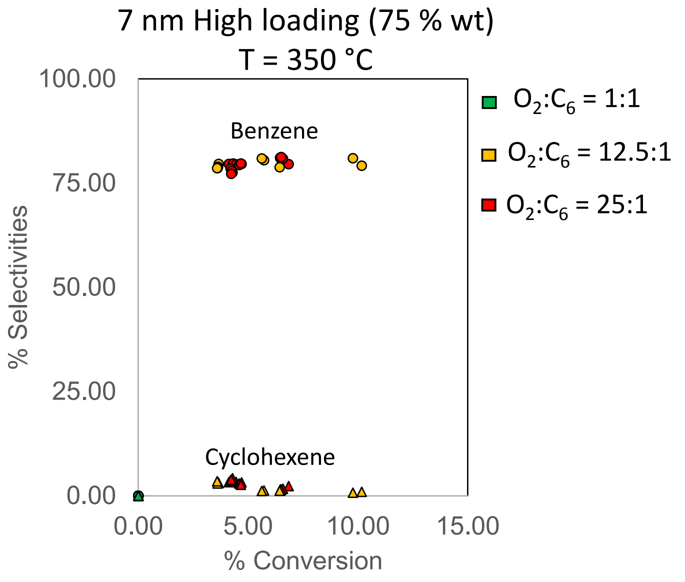

Fig. S6. Effect of O_2:Cyclohexane ratio on the cyclohexene and benzene selectivities on 7 nm $CoFe_2O_4$ particles with 75 % wt loading on rGO. Benzene (filled '○' symbols) and Cyclohexene (filled 'Δ' symbols)

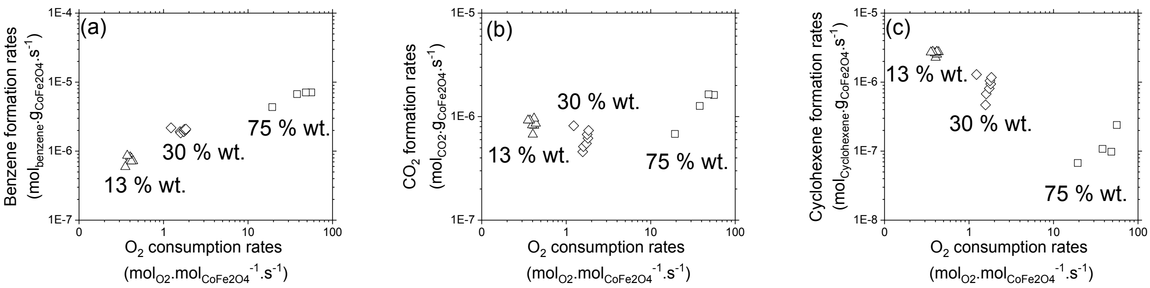

Fig. S7. Correlation between benzene, CO_2, and cyclohexene formation rates as a function of O_2 consumption rates for 7 nm particle at various % wt. loadings on RGO support.

Section S3.4. Resident time independent selectivity towards CO_2: Indication of autocatalysis

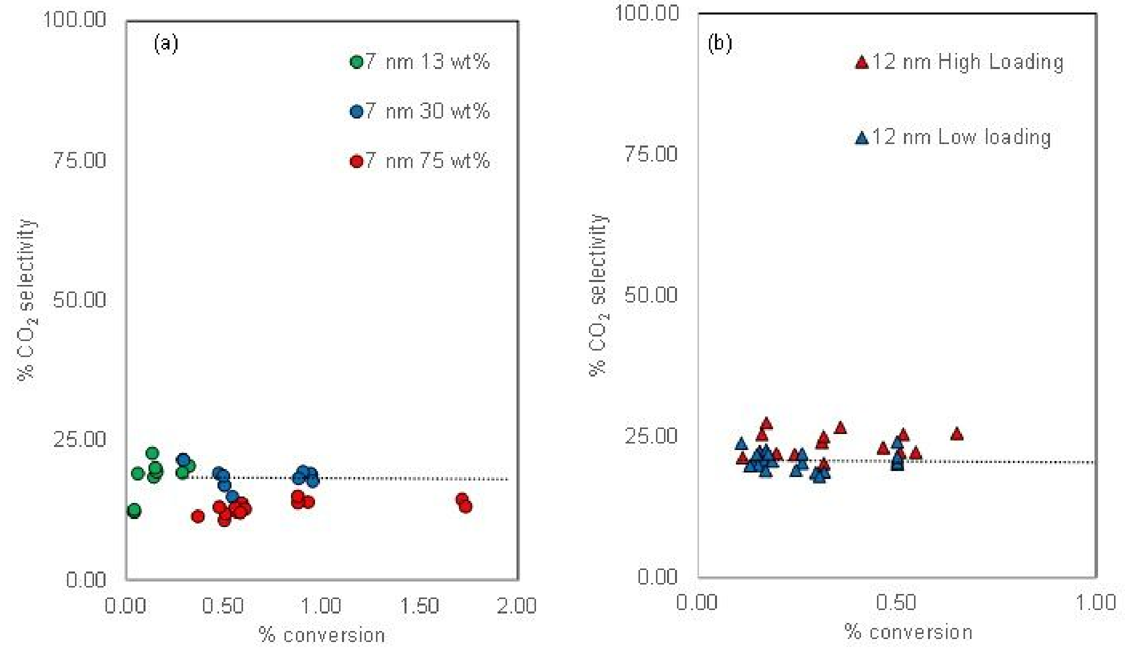

Fig S8. % CO_2 selectivity plotted as a function of % cyclohexane conversion at 350 °C and $P_{Cyclohexane}$ = P_{O2} = 0.2 kPa for 13 nm (a) and 6 nm (b) with various % wt loadings on rGO. The fractional conversion was varied by changing the reaction resident times. The insensitivity of CO_2 selectivity as a function of reaction resident times is reflected in Eqn. S20 and might suggest that CO_2 is formed on different active sites (probably OOH) via autocatalysis.

Section S3: High resolution XPS spectra of C 1s and O 1s photoelectrons of the 75% and 13 % wt loading $CoFe_2O_4$ fresh and spent catalysts

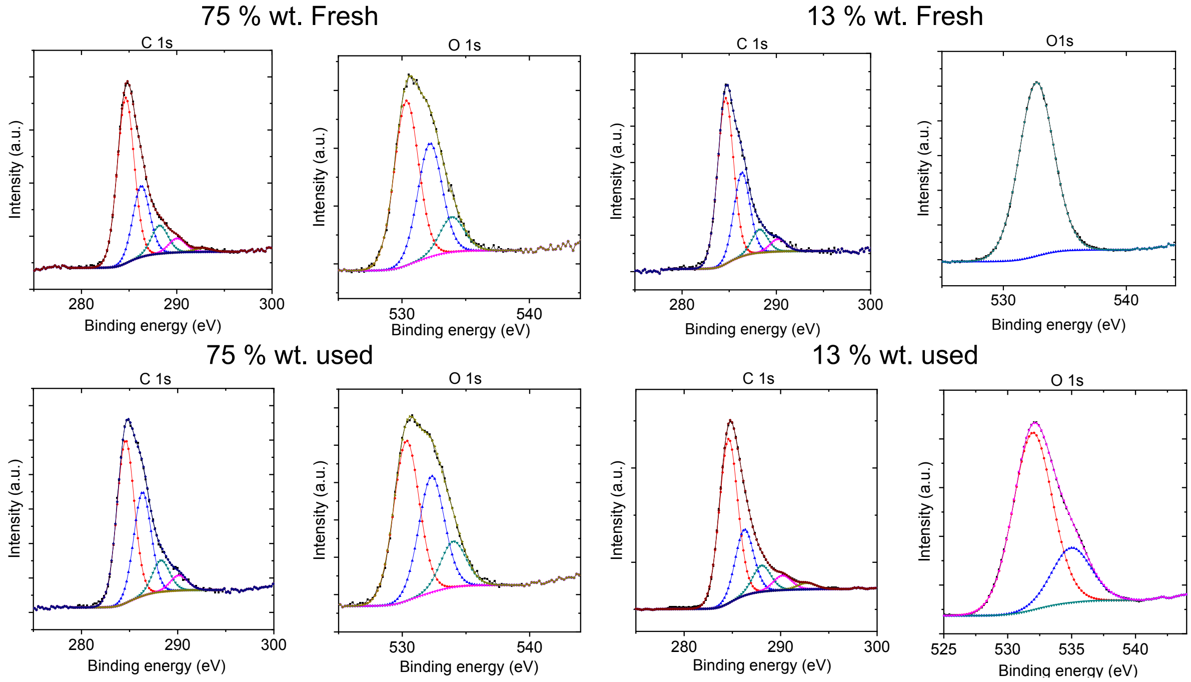

Fig. S9. Deconvoluted XPS high resolution spectra of C 1s and O 1s of the 75% and 13 % wt loading $CoFe_2O_4$ fresh and spent (after reaction 400 °C, P_{O2} = 0.2 kPa, $P_{Cyclohexane}$ = 0.2 kPa) catalysts powders.

Spectra of C 1s photoelectrons are composed of four components. The most intense component at 284.6 eV belongs to C-C bonds and the remaining peaks are assignable to oxygen containing functionalities [6] (e.g. binding energy of component 286.2 eV is characteristic of C-O bond, peak at 288.0 eV for C=O bond) . Oxygen 1s spectra taken from samples with a loading of 75% are comprised of peaks centered at 530.3, 532.2 and 533.9 eV. The low binding energy peak is associated with lattice oxygen of ferrite, the remaining peaks come from oxygen containing funtional groups on graphene oxide. The peak at 532.2 eV is usually assigned to C=O bond and the peak at 533.9 eV to the carboxyl group. For samples with a loading 13 % wt. due to low concentration of cobalt ferrite spectrum of O 1s electrons is dominated by components from graphene oxide. For a fresh sample with a loading 13 % wt. single peak fits well O 1s spectrum but the width (FWHM) of the peak is too broad for single oxygen species. Two or three peaks can be used with constraints on FWHM and peak separations but such a procedure may not be well substantiated.

Section S4: X-PEEM images and XAS spectra acquisition from individual particles

Fig. S10 (a) and (b) shows one of the XPEEM images recorded at the range of photon energies 700-730 eV in the case of Fe L3 edge to generate the sequence of the XAS spectra. The ratios spectral profile 1 / spectral profile 2 are used for further analysis using MCR ALS.

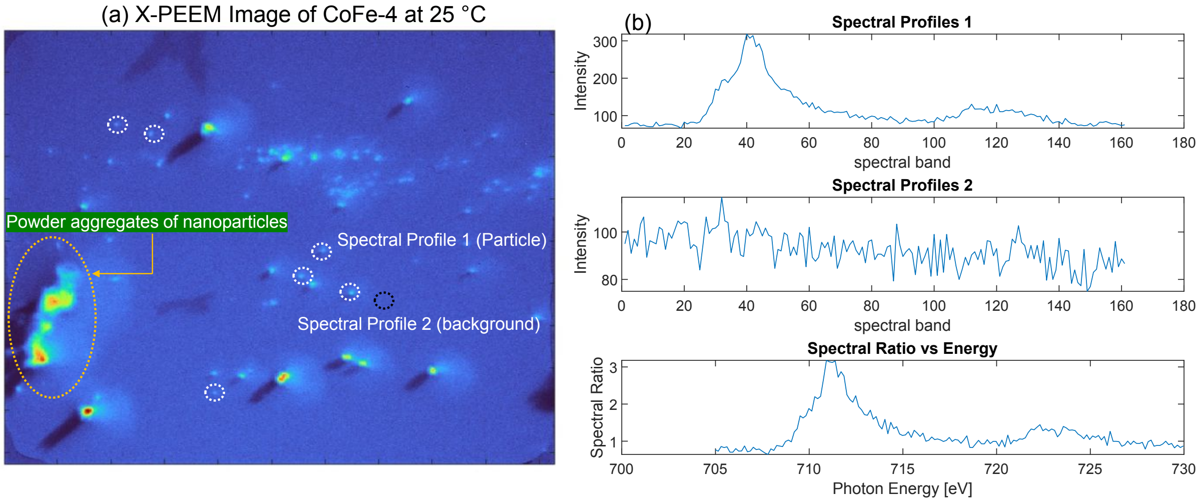

Fig. S10. (a) and (b) show an XPEEM image (25 °C) and the XA spectra acquired from individual particle aggregate (Spectral Profile 1, dotted white circle), background (Spectral Profile 2, dotted black circle) and resulting spectral ratio (Spectral Profile 1/ Spectral Profile 2). The dotted circles (white) show the particles areas selected for the spectra extraction. Spectral bands denote the XPEEM frame number recorded at each photon energy.

Section S5: Chemometric Analysis of XA spectra: PCA and MCRALS and comparison with the literature

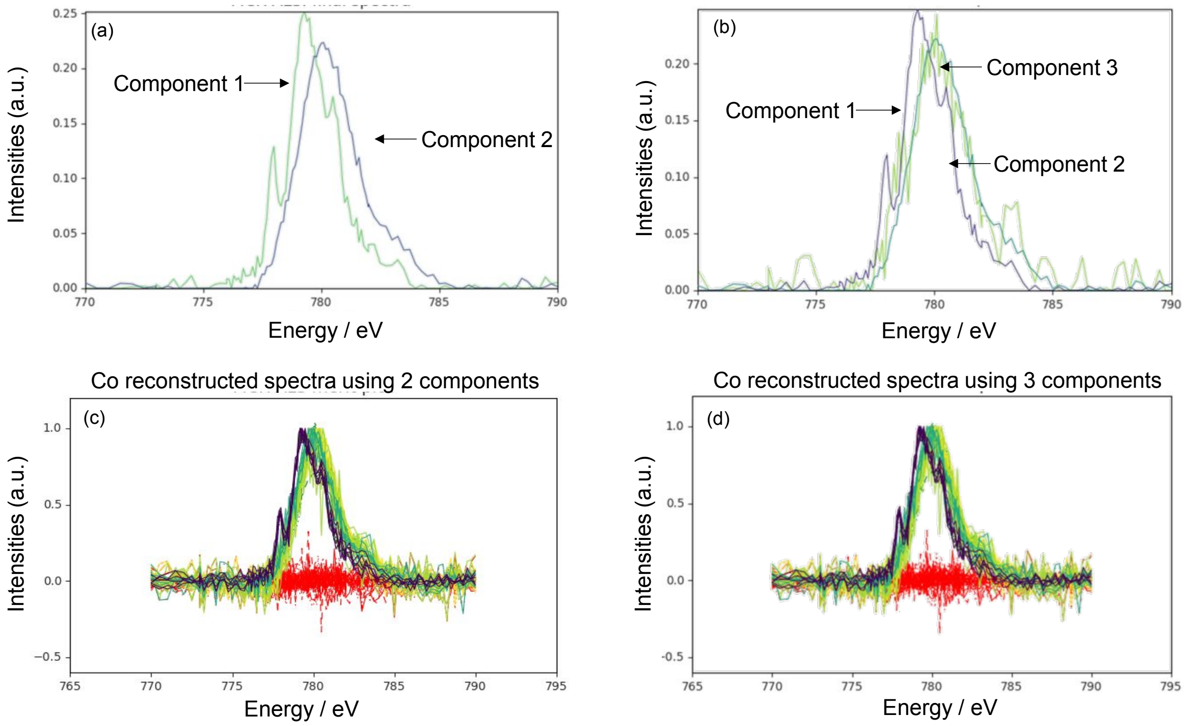

Fig. S11. XAS spectra of Co species reconstructed from MCR-ALS analysis using (a) two and (b) three components respectively on 13 nm CoFe2O4 particles. The third component in Fig. S3(b) completely overlaps with the second one suggesting only two components are enough to model the experimental Co spectra. Reconstruction of the experimental spectra from the pure species using (c) two and (d) three components. Table S3(e) overviews the percentage of variance explained per principal component (PC). Two components adequately explain the experimental spectra.

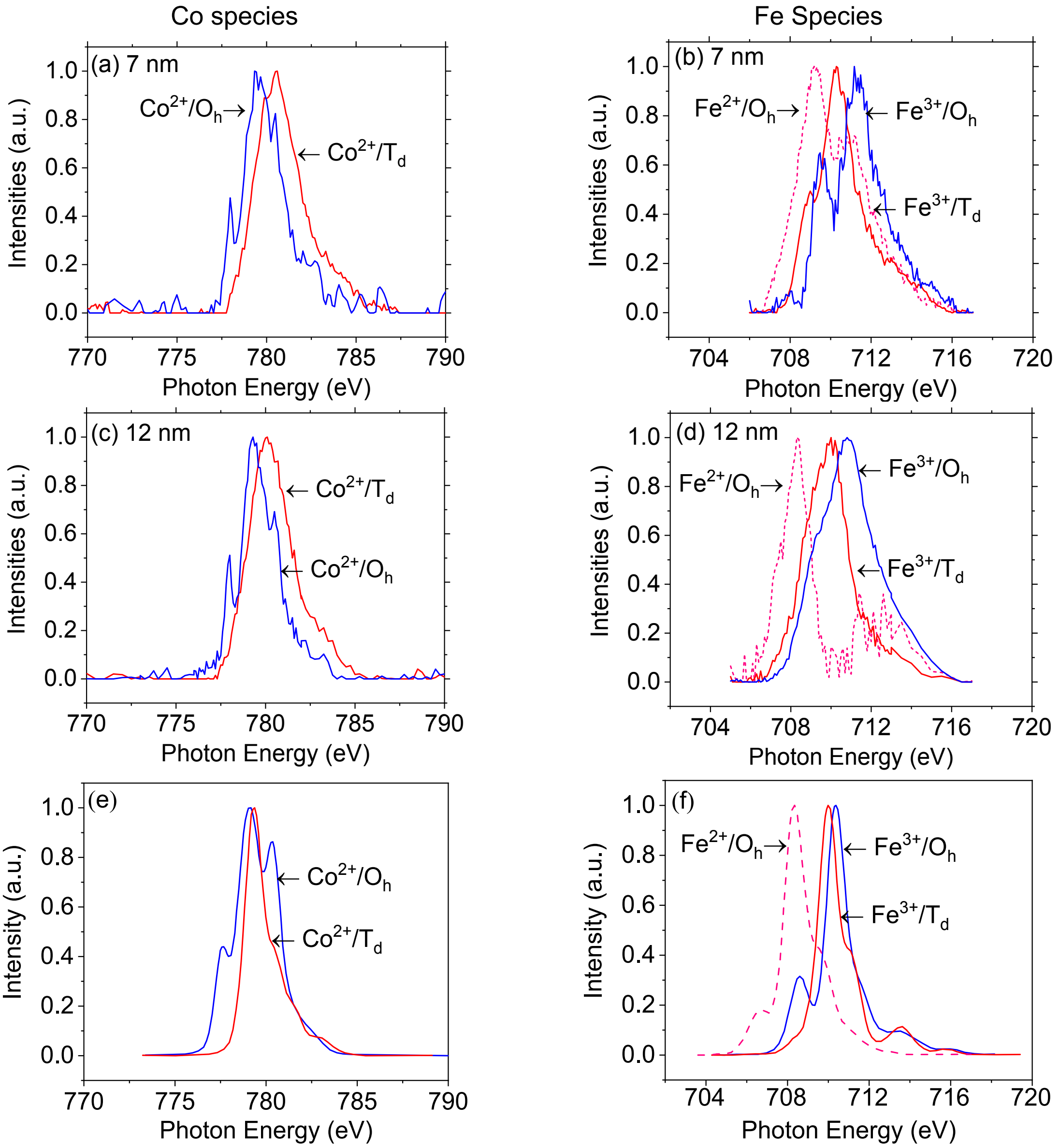

Fig. S12. Comparison between the XAS spectra of the pure species Co and for Fe obtained using MCR ALS (a) and (b) for 7 nm and (c) and (d) for 12 nm respectively, and corresponding theoretically calculated XA spectra of Co (e) and Fe (f) species by Moyer et al [7] using LFM model.

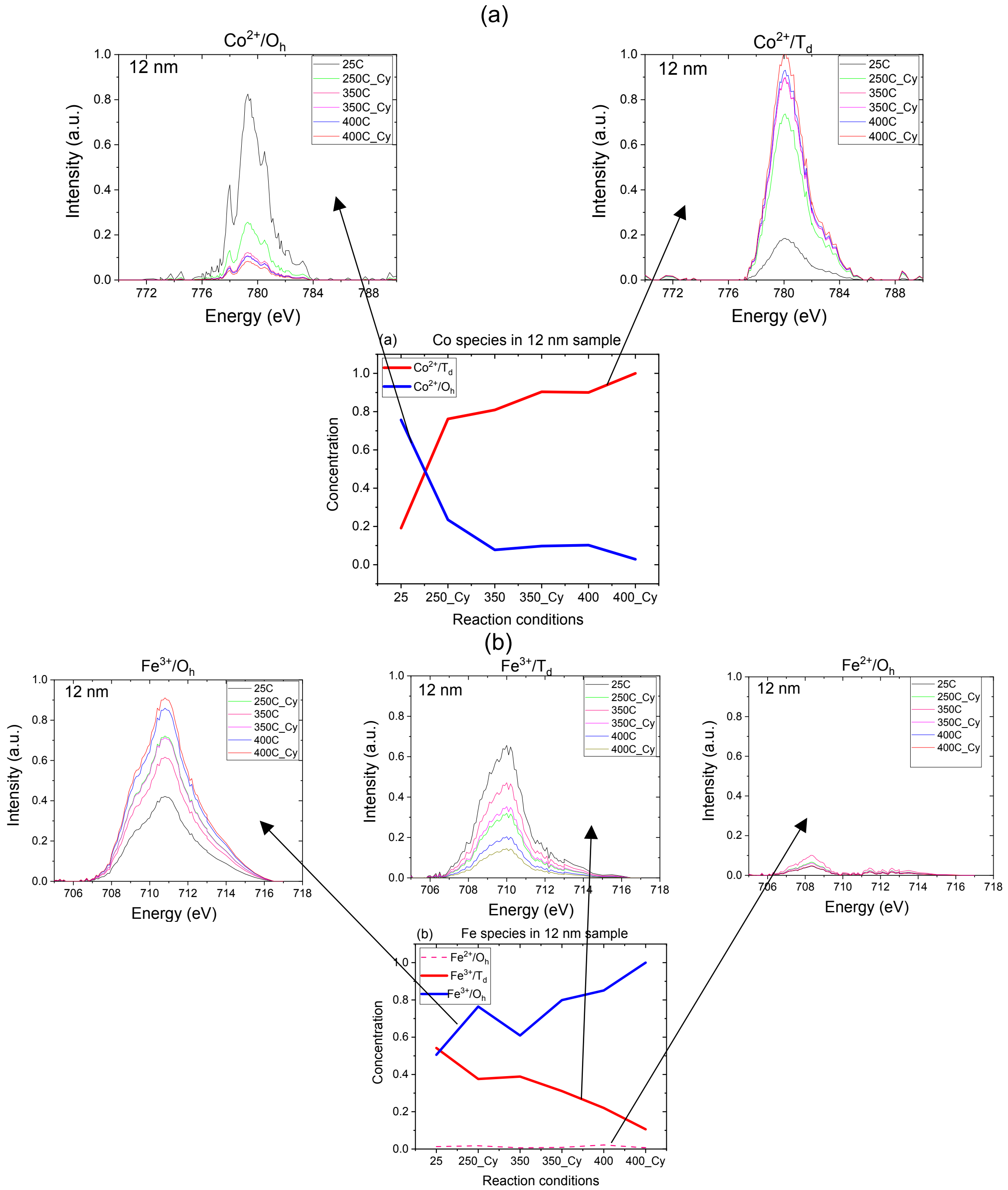

Fig. S13. Evolution of (a) Co^{2+} (T_d and O_h) and (b) Fe^{3+} (Td, Oh and Fe^{2+}/O_h) spectra of pure species as a function of reaction conditions. The spectra at each reaction conditions are average of 6 spectra (acquired from 6 different particle aggregates) for 12 nm particles on GO support (RGO-CoFe-4).

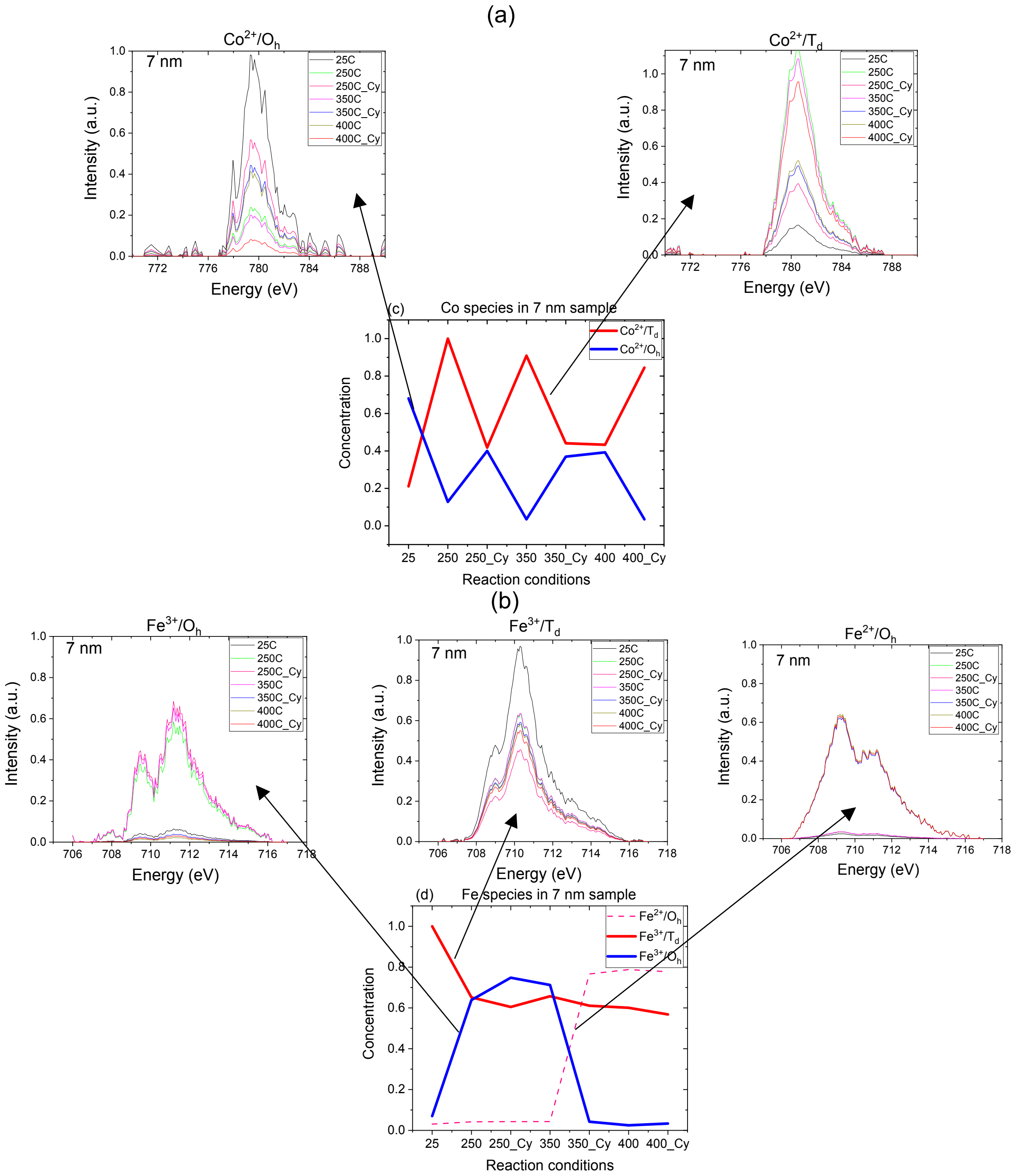

Fig. S14. Evolution of (a) Co^{2+} (T_d and O_h) and (b) Fe^{3+} (Td, Oh and Fe^{2+}/O_h) spectra of pure species as a function of reaction conditions. The spectra at each reaction conditions are average of 6 spectra (acquired from 6 different particle aggregates) for 7 nm particles on GO support (RGO-CoFe-4).

Section S6. Derivation of the rate expressions for cyclohexane oxidative dehydrogenation reactions and for the relations between primary dehydrogenation product selectivity ($S_{Cyclohexane}$) with the fractional cyclohexane conversion (X)

Rate Expressions:

Following the elementary steps in Scheme 1, the rate expressions can be derived for cyclohexene ($C_6^{=}$), benzene ($C_6^{3=}$) and CO_2 using the pseudo-steady state hypothesis (PSSH) on reactive surface intermediates, and from the assumptions about irreversible or quasi-equilibrated nature of elementary steps, as depicted in Scheme 1. From PSSH, the concentrations of the reactive oxygen derived species (O* adatoms) [O*], adsorbed cyclohexane upon these O* species [C_6—O*O*], and cyclohexane-derived adsorbed species after first C-H abstraction event [$C_6^{*=}$] are given by:

$$\frac{d[\mathrm{O}*]}{[L]dt} = \sqrt{\mathrm{K}_{O_2}} \cdot \sqrt{\mathrm{P}_{O_2}} - \mathrm{k}_{\mathrm{H_2O}} \cdot [\mathrm{H_2O}] \approx 0 \qquad \text{Eqn. S1}$$

$$\frac{d[\mathrm{C_6}\cdots\mathrm{O}*\mathrm{O}*]}{[L]dt} = k_{\mathrm{ads}} \cdot P_{\mathrm{C_6}}[\mathrm{O}*]^2 - k_{-\mathrm{ads}}[\mathrm{C_6}\cdots\mathrm{O}*\mathrm{O}*] \approx 0 \qquad \text{Eqn. S2}$$

$$\frac{d[C_6^{*=}]}{[L]dt} = k'_{C-H} \cdot [\mathrm{C_6}\cdots\mathrm{O}*\mathrm{O}*] - \mathrm{k}_{\mathrm{desorb}}[C_6^{*=}] - k''_{C-H}[C_6^{*=}] \approx 0 \qquad \text{Eqn. S3}$$

where, k_i and k_{-j} are the forward and reverse rate constants for each elementary step, respectively and P_{O2} and P_{C6} are the partial pressures of O_2 and cyclohexane respectively and the concentration of adsorbed species are denoted in squared brackets [ ]. Assuming that adsorbed O and cyclohexane species are in quasi-equilibrium with the respective gas phase, the steady-state surface concentrations can be given as

$$[\mathrm{O}*] = \frac{\sqrt{\mathrm{K}_{O_2}} \cdot \sqrt{\mathrm{P}_{O_2}}}{\mathrm{k}_{\mathrm{H_2O}} \cdot [\mathrm{H_2O}]} \qquad \text{Eqn. S4}$$

$$[\mathrm{C_6}\cdots\mathrm{O}*\mathrm{O}*] = \frac{K_{\mathrm{ads}} \cdot \mathrm{K}_{O_2} \cdot \mathrm{P}_{O_2} \cdot P_{\mathrm{C_6}}}{\mathrm{k}^2_{\mathrm{H_2O}} \cdot [\mathrm{H_2O}]^2} \qquad \text{Eqn. S5}$$

$$[C_6^{*=}] = \frac{k'_{C-H} \cdot K_{\mathrm{ads}} \cdot \mathrm{K}_{O_2} \cdot \mathrm{P}_{O_2} \cdot P_{\mathrm{C_6}}}{(\mathrm{k}^2_{\mathrm{H_2O}} \cdot [\mathrm{H_2O}]^2)(\mathrm{k}_{\mathrm{desorb}} + k''_{C-H})} \qquad \text{Eqn. S6}$$

The [OOH] concentration can be given by:

$$[OOH] = \mathrm{k}^2_{\mathrm{H_2O}}[\mathrm{H_2O}]^2[\mathrm{O}*]^2 = \mathrm{K}_{O_2} \cdot \mathrm{P}_{O_2} \qquad \text{Eqn. S7}$$

The rates of formation and consumption of cyclohexene ($r_{C^{=}_{6(g)}}$), benzene ($r_{C^{3=}_{6(g)}}$) and CO_2 along the catalyst bed are given by

$$\mathrm{r}_{C^{=}_{6(g)}} = \mathrm{k}_{\mathrm{desorb}}[C_6^{*=}] - \mathrm{k}'''_{\mathrm{C-H}} \cdot [\mathrm{O}*]^2 \cdot [C^{=}_{6(g)}] \qquad \text{Eqn. S8}$$

Substituting the values of $[C_6^{*=}]$ and $[\mathrm{O}*]$ and re-arranging we get,

$$\frac{\mathrm{r}_{C^{=}_{6(g)}}}{[L]} = \frac{\mathrm{k}_{\mathrm{desorb}} \cdot k'_{C-H} \cdot K_{\mathrm{ads}} \cdot \mathrm{K}_{O_2} \cdot \mathrm{P}_{O_2} \cdot P_{\mathrm{C_6}}}{(\mathrm{k}^2_{\mathrm{H_2O}} \cdot [\mathrm{H_2O}]^2)(\mathrm{k}_{\mathrm{desorb}} + k''_{C-H})} - \frac{\mathrm{k}'''_{\mathrm{C-H}} \cdot \mathrm{K}_{O_2} \cdot \mathrm{P}_{O_2} \cdot [C^{=}_{6(g)}]}{\mathrm{k}^2_{\mathrm{H_2O}} \cdot [\mathrm{H_2O}]^2} \qquad \text{Eqn. S9}$$

Similarly,

$$\frac{r_{C_{6(g)}^{3=}}}{[L]} = \frac{k''_{C-H}k'_{C-H} \cdot K_{ads} \cdot K_{O_2} \cdot P_{O_2} \cdot P_{C_6}}{(k^2_{H_2O} \cdot [H_2O]^2)(k_{desorb} + k''_{C-H})} + \frac{k'''_{C-H} \cdot K_{O_2} \cdot P_{O_2} \cdot [C^{=}_{6(g)}]}{k^2_{H_2O} \cdot [H_2O]^2} \quad \text{Eqn. S10}$$

Adding Eqn. S9 and S10 yields the rates of formation of $[C_6^{*=}]$ which is first order with respect to cyclohexane partial pressure, as observed in Fig. 12 in the main text. It also suggests, that $r_{C_6^{*=}}$ could also linearly depend on oxygen partial pressure, which we observed true for only high loading samples.

$$\frac{r_{C_6^{*=}}}{[L]} = \left(\frac{k'_{C-H} \cdot K_{ads} \cdot K_{O_2} \cdot P_{O_2}}{(k^2_{H_2O} \cdot [H_2O]^2)}\right) P_{C_6} \quad \text{Eqn. S11}$$

And rate of formation of CO_2 can be given as

$$\frac{r_{CO_2}}{[L]} = k_{C-C} K_{O_2} \cdot P_{O_2} P_{C_6} \quad \text{Eqn. S12}$$

Selectivity conversion expression:

The effects of cyclohexane conversion on cyclohexene and benzene selectivities are expressed here in the context of primary and secondary steps in Scheme 1 using the mathematical treatments appropriate for plug-flow reactors under conditions of low cyclohexane conversion (X < 0.02), for which the concentration of reactants remain essentially constant throughout the bed and the bed residence time (τ, s.[L]) can be approximately related to conversion (X) by (See Fig. S6)

$$\frac{r_{C^{=}_{6(g)}}}{[L]} = \frac{X}{\tau} \quad \text{Eqn. S13}$$

Relating Eqn. 13 to Eqn. 9, we get

$$\frac{X}{\tau} = \frac{r_{C^{=}_{6(g)}}}{[L]} = \frac{k'_{C-H} \cdot K_{ads} \cdot K_{O_2} \cdot P_{O_2} \cdot P_{C_6}}{(k^2_{H_2O} \cdot [H_2O]^2)(1 + \frac{k''_{C-H}}{k_{desorb}})} - \frac{k'''_{C-H} \cdot K_{O_2} \cdot P_{O_2} \cdot [C^{=}_{6(g)}]}{k^2_{H_2O} \cdot [H_2O]^2} \quad \text{Eqn. S14}$$

$$X = \frac{r_{C^{=}_{6(g)}}\tau}{[L]} = \left(\frac{k'_{C-H} \cdot K_{ads} \cdot K_{O_2} \cdot P_{O_2} \cdot P_{C_6}}{(k^2_{H_2O} \cdot [H_2O]^2)(1 + \frac{k''_{C-H}}{k_{desorb}})}\right)\tau - \left(\frac{k'''_{C-H} \cdot K_{O_2} \cdot P_{O_2} \cdot [C^{=}_{6(g)}]}{k^2_{H_2O} \cdot [H_2O]^2}\right)\tau \quad \text{Eqn. S15}$$

Following this approach and the elementary steps in Scheme 1, Eqn. S9 and S10 can be expressed using the molar concentrations of cyclohexane, cyclohexene, and benzene $[C_{6(g)}]$ $[C^{=}_{6(g)}]$ and $[C^{3=}_{6(g)}]$ as follows as their ordinary differential equations:

$$\frac{d[C^{=}_{6(g)}]}{dt} = \frac{k_{desorb} \cdot k'_{C-H} \cdot K_{ads} \cdot K_{O_2} \cdot P_{O_2} \cdot [C_{6(g)}]}{(k^2_{H_2O} \cdot [H_2O]^2)(k_{desorb} + k''_{C-H})} - \frac{k'''_{C-H} \cdot K_{O_2} \cdot P_{O_2} \cdot [C^{=}_{6(g)}]}{k^2_{H_2O} \cdot [H_2O]^2} \quad \text{Eqn. S16}$$

And

$$\frac{d[C^{3=}_{6(g)}]}{dt} = \frac{k''_{C-H}k'_{C-H} \cdot K_{ads} \cdot K_{O_2} \cdot P_{O_2} \cdot [C_{6(g)}]}{(k^2_{H_2O} \cdot [H_2O]^2)(k_{desorb} + k''_{C-H})} + \frac{k'''_{C-H} \cdot K_{O_2} \cdot P_{O_2} \cdot [C^{=}_{6(g)}]}{k^2_{H_2O} \cdot [H_2O]^2} \quad \text{Eqn. S17}$$

$$\frac{d[CO_2]}{dt} = k_{C-C} \cdot \mathrm{K}_{O_2} \cdot \mathrm{P}_{O_2} [C_{6\,(g)}] \quad \text{Eqn. S18}$$

The ordinary differential equations for the rates of formation and consumption of cyclohexene, benzene, and CO_2 with respect to the residence time (τ) along the catalyst bed is given by multiplying both sides by $\frac{\tau_0}{[C_{6\,(g)}]}$

$$\frac{\partial \emptyset'_{\mathrm{C6}}}{\partial \xi} = \frac{\tau_0 \cdot \mathrm{k_{desorb}} \cdot k'_{C-H} \cdot K_{\mathrm{ads}} \cdot \mathrm{K}_{O_2} \cdot \mathrm{P}_{O_2}}{(\mathrm{k}^2_{\mathrm{H_2O}} \cdot [\mathrm{H_2O}]^2)(\mathrm{k_{desorb}} + k''_{C-H})} - \tau_0 \frac{\mathrm{k}'''_{\mathrm{C-H}} \cdot \mathrm{K}_{O_2} \cdot \mathrm{P}_{O_2}}{\mathrm{k}^2_{\mathrm{H_2O}} \cdot [\mathrm{H_2O}]^2} \cdot \emptyset'_{\mathrm{C6}} \quad \text{Eqn. S18}$$

$$\frac{\partial \emptyset''_{\mathrm{C6}}}{\partial \xi} = \frac{\tau_0 \cdot k''_{C-H} \cdot k'_{C-H} \cdot K_{\mathrm{ads}} \cdot \mathrm{K}_{O_2} \cdot \mathrm{P}_{O_2}}{(\mathrm{k}^2_{\mathrm{H_2O}} \cdot [\mathrm{H_2O}]^2)(\mathrm{k_{desorb}} + k''_{C-H})} + \tau_0 \frac{\mathrm{k}'''_{\mathrm{C-H}} \cdot \mathrm{K}_{O_2} \cdot \mathrm{P}_{O_2}}{\mathrm{k}^2_{\mathrm{H_2O}} \cdot [\mathrm{H_2O}]^2} \cdot \emptyset'_{\mathrm{C6}} \quad \text{Eqn. S19}$$

$$\frac{\partial \emptyset_{CO_2}}{\partial \xi} = \tau_0 \cdot k_{C-C} \cdot \mathrm{K}_{O_2} \cdot \mathrm{P}_{O_2} = constant \quad \text{Eqn. S20}$$

where, $\emptyset'_{\mathrm{C6}} = \frac{[C^{=}_{6\,(g)}]}{[\mathrm{C}_{6\,(g)}]}$ is the ratio of molar concentration of cyclohexene and cyclohexane, $\emptyset''_{\mathrm{C6}} = \frac{[C^{3=}_{6\,(g)}]}{[\mathrm{C}_{6\,(g)}]}$ is the ratio of molar concentrations of benzene and cyclohexane, and $\xi = \frac{\tau}{\tau_0}$ with τ_0 being the resident time at initial low conversion.

The above equations can be simplified (S18 and S19) as follows:

Let's substitute

$$\alpha = \frac{k'_{C-H} \cdot K_{\mathrm{ads}} \cdot \mathrm{K}_{O_2} \cdot \mathrm{P}_{O_2}}{(\mathrm{k}^2_{\mathrm{H_2O}} \cdot [\mathrm{H_2O}]^2)(1 + \frac{k''_{C-H}}{\mathrm{k_{desorb}}})} \quad \text{Eqn. S21}$$

$$\beta = \frac{k''_{C-H} \cdot k'_{C-H} \cdot K_{\mathrm{ads}} \cdot \mathrm{K}_{O_2} \cdot \mathrm{P}_{O_2}}{(\mathrm{k}^2_{\mathrm{H_2O}} \cdot [\mathrm{H_2O}]^2)(\mathrm{k_{desorb}} + k''_{C-H})} \quad \text{Eqn. S22}$$

And

$$\gamma = \frac{\mathrm{k}'''_{\mathrm{C-H}} \cdot \mathrm{K}_{O_2} \cdot \mathrm{P}_{O_2}}{\mathrm{k}^2_{\mathrm{H_2O}} \cdot [\mathrm{H_2O}]^2} \quad \text{Eqn. S23}$$

Thus, we get,

$$\frac{\partial \emptyset'_{\mathrm{C6}}}{\partial \xi} = \alpha - \gamma \cdot \emptyset'_{\mathrm{C6}} \quad \text{Eqn. S24}$$

$$\frac{\partial \emptyset''_{\mathrm{C6}}}{\partial \xi} = \beta + \gamma \cdot \emptyset'_{\mathrm{C6}} \quad \text{Eqn. S25}$$

Fig. 13 (main text) shows that the cyclohexene selectivities decrease with fractional cyclohexane conversion in a linear manner at low conversion allowing Eqns. 24 and 25 to be solved by expanding the exponents using perturbation analysis (Taylor expansion) and solved asymptotically up to first order to yield:

$$\emptyset'_{C6} = \alpha \cdot \xi - \frac{\alpha \cdot \gamma \cdot \xi^2}{2} \qquad \text{Eqn. S26}$$

$$\emptyset''_{C6} = \frac{\alpha \cdot \gamma \cdot \xi^2}{2} + \beta \cdot \xi \qquad \text{Eqn. S27}$$

$$\emptyset_{CO_2} = 0 \qquad \text{Eqn. S28}$$

We further express the fractional cyclohexane conversion as follows:

$$X_{fractional} = \emptyset'_{C6} + \emptyset''_{C6} + \emptyset_{CO_2} = (\alpha + \beta) \cdot \xi \qquad \text{Eqn. S29}$$

The cyclohexene selectivity then can be expressed as

$$S_{C_{6(g)}^{=}} = \frac{\emptyset'_{\mathbf{C6}}}{\emptyset'_{\mathbf{C6}} + \emptyset''_{\mathbf{C6}} + \emptyset_{CO_2}} \qquad \text{Eqn. S30}$$

$$S_{C_{6(g)}^{=}} = \frac{\alpha}{\alpha + \beta} - \frac{\alpha \cdot \gamma \cdot \xi}{2 \cdot (\alpha + \beta)} \qquad \text{Eqn. S31}$$

Substituting the values of α, β and γ from Eqn. S21, S22 and S23 we get

$$S_{C_{6(g)}^{=}} = \frac{1}{\left(1 + \frac{k''_{C-H}}{k_{desorb}}\right)} - X_{fractional}\left(\frac{1}{1 + \frac{k''_{C-H}}{k_{desorb}}}\right)\frac{k'''_{C-H}}{2 \cdot k'_{C-H} \cdot K_{ads}} \qquad \text{Eqn. S32}$$

The functional form of the equation S32 is used to fit all the data in Fig. 13 (main text). The chi-square minimization method is used to minimize the deviations from the theoretical curve (function curve) from the experimental points in Fig. 13 using number of iterations to adjust the parameter values (in this case, ratios of $\frac{k''_{C-H}}{k_{desorb}}$ and $\frac{k''_{C-H}}{k'_{C-H}}$ Eqn. 32).

$$X^2 = \sum_{i=1}^{n} \frac{(Observed_i - Expected_i)^2}{Expected_i} \qquad \text{(Eqn.S33)}$$

Figure S12 shows the parity plot for measured selectivity values plotted against those determined from the regression of all the data in Fig. 13 (main text).

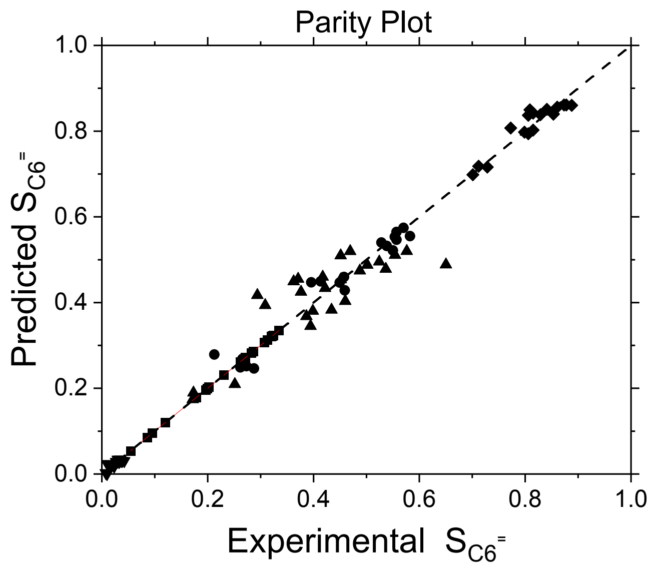

Fig. S15. Parity plot of measured selectivities ($S_{C6^=}$) and those determined by regression of all the data in Fig. 13 to the functional form of Eqn. 32 at 350 °C and $P_{Cyclohexane} = P_{O2}$ = 0.2 kPa. (◆) 7 nm 13 % wt, (●) 7 nm 30 % wt, (▼) 7 nm 75 % wt, (▲) 12 nm 26 % wt and (▪) 12 nm 76 % wt samples.

References:

1. Llenas, M.; Sandoval, S.; Costa, P. M.; Oró-Solé, J.; Lope-Piedrafita, S.; Ballesteros, B.; Al-Jamal, K. T.; Tobias, G., Microwave-Assisted Synthesis of SPION-Reduced Graphene Oxide Hybrids for Magnetic Resonance Imaging (MRI). *Nanomaterials* **2019,** *9* (10), 1364.
2. Bertran, A.; Sandoval, S.; Oró-Solé, J.; Sánchez, À.; Tobias, G., Particle size determination from magnetization curves in reduced graphene oxide decorated with monodispersed superparamagnetic iron oxide nanoparticles. *Journal of Colloid and Interface Science* **2020,** *566*, 107-119.
3. Tyo, E. C.; Yin, C.; Di Vece, M.; Qian, Q.; Kwon, G.; Lee, S.; Lee, B.; DeBartolo, J. E.; Seifert, S.; Winans, R. E.; Si, R.; Ricks, B.; Goergen, S.; Rutter, M.; Zugic, B.; Flytzani-Stephanopoulos, M.; Wang, Z. W.; Palmer, R. E.; Neurock, M.; Vajda, S., Oxidative Dehydrogenation of Cyclohexane on Cobalt Oxide (Co3O4) Nanoparticles: The Effect of Particle Size on Activity and Selectivity. *ACS Catalysis* **2012,** *2* (11), 2409-2423.
4. Lachkov, P. T.; Chin, Y.-H., Catalytic consequences of reactive oxygen species during C3H6 oxidation on Ag clusters. *Journal of Catalysis* **2018,** *366*, 127-138.
5. Chin, Y. H.; Buda, C.; Neurock, M.; Iglesia, E., Reactivity of chemisorbed oxygen atoms and their catalytic consequences during CH4-O2 catalysis on supported Pt clusters. *J Am Chem Soc* **2011,** *133* (40), 15958-78.
6. Kovtun, A.; Jones, D.; Dell'Elce, S.; Treossi, E.; Liscio, A.; Palermo, V., Accurate chemical analysis of oxygenated graphene-based materials using X-ray photoelectron spectroscopy. *Carbon* **2019,** *143*, 268-275.
7. Moyer, J. A.; Vaz, C. A. F.; Negusse, E.; Arena, D. A.; Henrich, V. E., Controlling the electronic structure of $Co_{(1-x)}$ $Fe_{(2+x)}$ O_4 thin films through iron doping. *Physical Review B* **2011,** *83* (3), 035121.
